# Elevated KNSTRN as a potential indicator for triple-negative breast cancer progression and immune infiltration

**DOI:** 10.3389/fimmu.2025.1572359

**Published:** 2025-10-23

**Authors:** Yurong Song, Yuxi Liu, Xiao Liu, Minfang Qi, Zhidong Sun, Yuan Cao

**Affiliations:** ^1^ Department of Basic Medical Sciences, The 960th Hospital of PLA, Jinan, China; ^2^ Department of Pathology, Affiliated Hospital of Guilin Medical University, Guilin, China

**Keywords:** KNSTRN, TNBC, prognosis, immune infiltration, tumor progression

## Abstract

**Background:**

Kinetochore localized astrin/SPAG5 binding protein (KNSTRN) is a protein-coding gene pivotal for the mitotic spindle’s operation, ensuring accurate chromosome separation and transition into anaphase. Existing literature indicates that it is associated with a variety of cancers. However, there is a lack of research to confirm that it is related to the malignant phenotype and immune infiltration of triple-negative breast cancer (TNBC). The objective of this study was to ascertain the potential role of KNSTRN in TNBC prognosis, immune infiltration and progression.

**Methods:**

We analyzed KNSTRN expression in TNBC using RNA-seq and single-cell transcriptome data from TCGA, GEO, and METABRIC datasets, correlating it with clinical features, prognosis, and immune infiltration. Functional enrichment analyses identified pathways regulated by KNSTRN in TNBC. *In vitro* siRNA knockdown in TNBC cell lines (MDA-MB-231 and BT549) assessed its impact on proliferation, migration, and DNA synthesis. RNA-seq was performed on BT549 cells with KNSTRN knockdown to validate the findings from the bioinformatic analysis. Immunohistochemistry was used to validate KNSTRN expression in tissue of patients with TNBC and other subtypes of breast cancer (Non-TNBC), as well as the association of KNSTRN expression and CD8+ T cell infiltration in TNBC.

**Results:**

KNSTRN was significantly overexpressed in TNBC compared to those in other breast cancer subtypes and normal tissues. High expression of KNSTRN is associated with a poor prognosis in TNBC. Functional enrichment analysis revealed that KNSTRN-associated differentially expressed genes (DEGs) were involved in cell cycle regulation, metabolism, and immune response pathways. Immune infiltration analysis showed that high KNSTRN expression was associated with reduced infiltration of CD8+ T cells. *In vitro* experiments confirmed that KNSTRN knockdown inhibited TNBC cell proliferation and migration. RNA-seq on BT549 cells with KNSTRN knockdown also validated that KNSTRN played a role in promoting cell cycle progression and cell proliferation.

**Conclusions:**

KNSTRN is a candidate biomarker for TNBC prognosis and a potential target for immunotherapeutic strategies. Its overexpression in TNBC is associated with aggressive tumor behavior and an immunosuppressive microenvironment, highlighting its significance in TNBC pathogenesis and prognosis.

## 1 Introduction

Breast cancer represents the most prevalent cancer among women and is the leading cause of cancer-related mortality. In 2022, approximately 9.66 million cases were reported, positioning it as the second most diagnosed cancer globally, accounting for 11.6% of all new cancer cases (1). Although some progress has been made in surgery, radiotherapy, chemotherapy and endocrine therapy in recent years, the prognosis of triple-negative breast cancer (TNBC) patients remains poor (2). TNBC represents a highly aggressive subtype of breast cancer, distinguished by the lack of estrogen receptor (ER), progesterone receptor (PR), and human epidermal growth factor receptor 2 (HER2) expression. TNBC constitutes a significant challenge in oncology due to its high recurrence rate and limited treatment options. Primarily affecting premenopausal women under 40 years of age, TNBC represents about 15–20% of all patients and is associated with worse prognosis in actual clinical scenarios (3) (4). In comparison to other subtypes, TNBC patients exhibit shorter survival times and higher mortality rates, with a 40% risk of death within five years of diagnosis (5). TNBC is characterized by its highly aggressive behavior, with approximately half of patients experiencing distant metastases, which significantly reduces the median survival time to 13.3 months post-metastasis (6). The prognosis for recurrent cases is particularly poor, with a mortality rate reaching 75% within the first three months. Due to its distinctive molecular signature, TNBC exhibits inherent resistance to hormonal interventions, rendering chemotherapy the principal therapeutic strategy, albeit with modest therapeutic outcomes (7).

TNBC is highly aggressive and heterogeneous, with a lack of effective therapeutic targets, leading to poor patient prognosis (8). Studies have revealed that the tumor immune microenvironment (TIME) plays a critical role in the development, progression, and treatment response of TNBC (9). The TIME in TNBC is often enriched with tumor-infiltrating lymphocytes, particularly CD8+ T cells, which are a key effector population capable of directly killing tumor cells and are associated with improved response to immunotherapy (10, 11). This microenvironment also contains immunosuppressive components such as regulatory T cells (Tregs), myeloid-derived suppressor cells (MDSCs), as well as inhibitory signaling pathways (such as PD-1/PD-L1 and CTLA-4) that compromise effective immune responses and lead to CD8+ T cell exhaustion (12). In-depth analysis of the TIME helps distinguish immune-activated from immune-suppressed subtypes, provides predictive biomarkers for response to immune checkpoint inhibitors and guides the development of combination therapies. Therefore, deciphering the composition and dynamics of the TIME in TNBC is crucial not only for understanding mechanisms of drug resistance and disease evolution, but also for informing novel immunotherapeutic strategies and improving clinical outcomes.

Kinetochore localized astrin/SPAG5 binding protein (KNSTRN) is a protein coding gene that encodes a kinetochore-associated protein critical for accurate chromosome segregation during mitosis (13). KNSTRN was highly concentrated on kinetochores from late prometaphase to anaphase and plays a pivotal role in ensuring proper cell division (5). Recent research has identified KNSTRN as a potential oncogene involved in the progression of various cancers, including cutaneous squamous cell carcinoma (14), hepatocellular carcinoma (15), lung adenocarcinoma (16, 17). High expression of KNSTRN is indicative of an unfavorable outcome due to its contribution to promoting cell cycle progression and tumor cell proliferation. Additionally, previous research has shown that KNSTRN may play a significant role in modulating the tumor immune microenvironment. Studies across pan-cancer datasets indicate that high KNSTRN expression is correlated with poor prognosis and alterations in immune cell infiltration (18). Specifically, KNSTRN upregulation is linked to increased infiltration of immunosuppressive cells such as regulatory T cells (Tregs), M2 macrophages, and myeloid-derived suppressor cells (MDSCs), while negatively correlating with cytotoxic CD8^+^ T cells and activated natural killer (NK) cells. In lung adenocarcinoma, high KNSTRN levels are positively associated with Th2 cells and markers of T-cell exhaustion (including PD-1, CTLA-4, and LAG-3) (15). Similarly, in hepatocellular carcinoma, KNSTRN upregulation correlates strongly with increased infiltration of regulatory T-cells (Tregs) and elevated expression of exhaustion markers such as PDCD1 and CTLA4 (16). Furthermore, KNSTRN expression correlates with resistance to immunotherapy and various chemotherapeutic agents, possibly through pathways involving endoplasmic reticulum stress (18, 19). Single-nucleotide variants in KNSTRN have been linked to adverse outcomes, highlighting its role as a potential indicator for diagnosis and a target for therapeutic interventions (20). Nevertheless, the role of KNSTRN in TNBC, particularly its implications for the prognosis, immune infiltration, tumor progression, and underlying mechanisms remains unclear.

In this study, we aimed to elucidate the role of KNSTRN in TNBC by integrating multiple analytical approaches. We conducted comprehensive analyses of RNA-seq and single-cell transcriptome data sourced from TCGA, GEO, and METABRIC databases to investigate the correlation between KNSTRN expression and clinical features, prognosis, and immune infiltration in TNBC. Additionally, we employed siKNSTRN in TNBC cell lines to assess its effects on cell proliferation, migration, and DNA synthesis. To validate the findings from the bioinformatic analysis, we conducted RNA-seq on BT549 cells after siKNSTRN transfection. Immunohistochemistry (IHC) was performed to evaluate KNSTRN expression in tumor and normal adjacent tissues (NAT) from TNBC patients and other breast cancer subtypes, as well as the correlation between KNSTRN expression and CD8+ T cell infiltration in TNBC.

## 2 Material and methods

### 2.1 Data preparation and processing

Clinical information and RNA expression data from tumor and normal tissues were sourced from Genotype-Tissue Expression (GTEx, http://www.gtexportal.org) databases and The Cancer Genome Atlas (TCGA, https://tcga-data.nci.nih.gov/tcga/). The UCSC XENA platform (https://xenabrowser.net/datapages/) was utilized for conducting pan-cancer analysis and generating Receiver Operating Characteristic Curve (ROC) curves. RNA-seq data were converted to TPM format and analyzed following the guidelines of TCGA. The study utilized the transcriptome sequencing data of 360 TNBC cases from SRP157974 in the European Nucleotide Archive database. The METABRIC breast cancer dataset was sourced from the cBioPortal, encompassing microarray data for 2509 cases of primary tumor tissues including TNBC cases. By downloading the TNBC dataset GSE76250 from the GEO database, this study utilized 165 cases of TNBC primary tumor tissues and 33 cases of adjacent normal tissues included in the dataset, comprising 33 pairs of matched TNBC tumor and adjacent non-tumor samples.

### 2.2 ROC analysis

The RNA-seq data for 33 common cancer including breast cancer were analyzed using the Wilcoxon rank sum and signed rank tests to identify variations in KNSTRN expression levels across multiple group comparisons and within paired samples. Utilizing the Wilcoxon rank sum test, we investigated the diagnostic value of KNSTRN expression levels in predicting a range of clinicopathological features including ER status, PR status, HER2 status, PAM50. The PAM50 classification data was obtained from the study conducted by Berger et al. (21). The cutoff to discriminate between TNBC and Non-TNBC was calculated by ROC analysis and Youden index calculation.

### 2.3 Survival analysis

To evaluate the prognostic relevance of KNSTRN expression levels in TNBC, we conducted Cox regression and Kaplan-Meier survival analysis by employing R software at version 4.4.2 equipped with the “survival” and “survminer” packages. The median expression level of KNSTRN was used as the threshold. The relationship between KNSTRN expression levels and relapse free survival (RFS) was investigated.

### 2.4 Functional enrichment analysis

Patients with TNBC from the METABRIC cohort were categorized into two groups based on the expression of KNSTRN. Differential genes between KNSTRN-low and KNSTRN-high groups were analyzed using the linear model from the limma package (22). The hallmark gene sets (23) derived from MSigDB (version 7.4) were utilized to conduct Gene Set Variation Analysis (GSVA) and Gene Set Enrichment Analysis (GSEA) (24). The enrichment significance of the hallmark signature in GSVA was also assessed using the limma package (22).

### 2.5 Immune infiltration analysis

We utilized the ssGSEA implemented with the GSVA package in R to investigate the association between immune cell infiltration and KNSTRN (24). The marker genes for these immune cell types were sourced from the study by Bindea G et al. (25). Following that, we examined how these immune cell types are distributed within tumors by employing seven alternative algorithms, including the ssGSEA, ESTIMATE (26), ConsensusTME (27), MCP-counter (28), EPIC (29), quanTIseq (30), and TIMER (31). To assess the association between KNSTRN expression and immune cell infiltration, we employed the Spearman correlation analysis. Furthermore, we applied the Wilcoxon rank-sum test to examine the differences in immune cell infiltration between the high and low KNSTRN expression groups. Next, analysis of the scRNA-seq data (GSE176078) was conducted using R software at version 4.4.2 along with the Seurat package (32). Canonical correlation analysis (33) was employed to integrate multiple single-cell samples, using the foremost 20 principal components for uniform manifold approximation and projection-based dimensionality reduction and the creation of a Shared Nearest Neighbor (SNN) graph. Cell clustering analysis was conducted using the Louvain algorithm method at a resolution setting of 0.8.

### 2.6 Cell culture

The human TNBC cell line MDA-MB-231 and BT549 was sourced from the BeNa Culture Collection. MDA-MB-231 was cultured in dulbecco’s modified eagle medium (DMEM) with high glucose and completed with 10% FBS and 1% penicillin-streptomycin. BT549 was cultured in RPMI1640 completed with 10% FBS, 1% penicillin-streptomycin and 10 μg/mL insulin. All cells were maintained in an incubator set to 37 °C and 5% CO_2_ and only cells with passage number under 10 were used for further experiments. Mycoplasma testing was routinely performed with Mycoalert Mycoplasma detection kit (Lonza) and no mycoplasma contamination was detected in any of the cultures.

### 2.7 RNA interference and transfection

The cells were placed in six-well plates, permitted to settle until they achieved 50% confluency. siKNSTRN transfection of MDA-MB-231 cells was carried out with the jetPRIME^®^ system (Polyplus, New York, NY, United States), adhering to the provided manufacturer’s procedures, meanwhile, a negative control siRNA was used for comparison. The cells were incubated after transfection for 24–48 hours to ensure efficient knockdown of KNSTRN expression.

### 2.8 RNA-seq

Transcriptome sequencing was performed on BT549 cells following transfection with siKNSTRN or siControl. Both the siKNSTRN group and the siControl group included three biological replicates. Total RNA was extracted and strand-specific libraries were prepared using poly-T magnetic beads for mRNA enrichment. Fragmented mRNA was reverse-transcribed into cDNA using random hexamers, with dUTP incorporated during second-strand synthesis to maintain strand orientation. After USER enzyme digestion to remove uracil-containing strands, libraries underwent end repair, A-tailing, adapter ligation, size selection, and PCR amplification. Library quality was assessed using Qubit, real-time PCR, and Bioanalyzer. Sequencing was carried out on an Illumina platform, and raw reads were processed with fastp for quality control. HISAT2 aligned clean reads to the reference genome, and featureCounts quantified gene expression in FPKM. Differential expression analysis was performed using the DESeq2 R package. Genes with |log2FoldChange | ≥ 1 and p-value < 0.05 were considered statistically significant DEGs. We used clusterProfiler R package for GO function enrichment and KEGG pathway enrichment analysis. When P < 0.05, it is considered that the GO or KEGG function is significantly enriched.

### 2.9 Quantitative real-time PCR

Cells were processed to extract total RNAs using TRIzol reagent (R0016, Invitrogen). The concentration and optical density (OD) of the RNA samples were assessed with the Nano-1000D microspectrophotometer. For mRNA analysis, 2 μg of RNA was utilized to generate cDNA through the Script Reverse Transcription Reagent Kit (RR047A, TaKaRa, Japan). The quantitative real-time PCR (qRT–PCR) was performed with the TB Green^®^ Premix Ex Taq™ II (CN830b, TaKaRa, Japan) on the SLAN-96P Real-time PCR System (HONGSHI, China). The thermal cycling profile consisted of 30 seconds at 95 °C, followed by 5 seconds at 90 °C and 10 seconds at 60 °C, repeated for a total of 40 cycles. The relative expression levels of the target genes were calculated using the 2-ΔΔCT method, with ACTB used as the reference gene. The primers sequences (Sangon Biotech, Shanghai, China) were as follows: KNSTRN forward primer, 5’-GCTACTGACACTGCCACCAGAA-3’; KNSTRN reverse primer, 5’- GCAACTGCTTGTTGACGGCTTC -3’; GAPDH forward primer, 5’-GTCTCCTCTGACTTCAACAGCG-3’; GAPDH reverse primer, 5’-ACCACCCTGTTGCTGTAGCCAA-3’; ACTB forward primer, 5’-CACCATTGGCAATGAGCGGTTC-3’; ACTB reverse primer, 5’-AGGTCTTTGCGGATGTCCACGT-3.

### 2.10 Western blotting

Cellular proteins were harvested using a RIPA buffer (Servicebio, Wuhan, China) enriched with both protease and phosphatase inhibitors. Concentrations of protein were ascertained utilizing the BCA protein assay kit (Solarbio, Beijing, China), followed by equalized protein separation on a 10% SDS-PAGE gel. Subsequently, the proteins were directed onto PVDF membranes (Millipore, Darmstad, Germany) using a constant voltage (100 V) for approximately 60 minutes. Post a 2-hour blocking period with 5% milk, the membranes were exposed to primary antibodies for an overnight period at a temperature of 4 °C. Once unbound primary antibodies were washed off three times, the membranes underwent a 1-hour incubation with secondary antibodies at room temperature. The KNSTRN antibody (26189-1-AP, 1:1000) and GAPDH antibody (60004-1-Ig, 1:10000) were employed as primary antibodies. Following another round of washing, protein bands were detected through an imaging system and the comparative expression levels of the target protein were determined using ImageJ software.

### 2.11 Wound healing assay

Cells were inoculated in six-well plates at a concentration of 1×10^5^ cells per well and permitted to reach 80% confluency to siKNSTRN. Subsequently, the cell monolayer was wounded with a 200 μL pipette tip. Images of the scrape were recorded under a microscope (magnification, ×10) at 0 hours (immediately after scratching), 24 hours, and 48 hours. The width of the gap was determined through the application of ImageJ software.

### 2.12 Transwell migration assay

The 12-well plates were equipped with transwell inserts that have an 8.0 μm pore size. The lower chamber received 1 mL of medium with 10% FBS to attract the cells. After incubation at 37 °C, the transwell inserts were carefully removed, excess medium was discarded, and non-migrated cells on the top surface were gently wiped off with a wet cotton swab. The cells that migrated to the bottom surface of the inserts were stabilized with 4% paraformaldehyde and dyed with a 0.5% crystal violet solution for 10 minutes at room temperature. The migration of cells was observed under a microscope and five random fields at 100× magnification were selected to count and analyze the number of cells that had passed through the pores.

### 2.13 EdU assay

Post-transfection with siKNSTRN or control siRNA, MDA-MB-231 cells were collected 24 hours later and plated into 24-well plates. The following day, the experiment proceeded with the EdU assay utilizing the EdU kit (C10310-1, RiboBio, Guangzhou, China). The cells were exposed to 50 µM EdU, a thymidine analog that incorporates into actively proliferating cells during DNA synthesis. After 2 hours of incubation, fixed cells were stained using Apollo staining solution for detecting incorporated EdU and Hoechst 33342 staining solution for staining the nuclei of all cells. Fluorescence microscopy was used to capture images, and the proliferation rate was evaluated by ImageJ based on the ratio of EdU-positive to total cells.

### 2.14 Immunohistochemistry

In this study, we conducted IHC using tissue microarrays to evaluate the expression of KNSTRN in breast cancer samples. Patient samples were derived from residual paraffin-embedded tissues following clinical pathological diagnosis (TNBC or other subtypes of breast cancer), which were then processed into tissue microarrays and sectioned for analysis. This part has been approved by the Ethics Committee of the 960th Hospital of the PLA (No 2024-112), and informed consent was waived. Immunohistochemical staining on tissue microarrays was performed as follows. Paraffin sections were baked and deparaffinized by immersing in fresh xylene for 5 minutes, followed by hydration with graded ethanol, and rinsing with distilled water. For antigen retrieval, slides were placed under high pressure with a pH 6.0 retrieval solution (C1032, Solarbio, CN). Endogenous peroxidase was blocked by incubating the slides in blocking solution for 15 minutes, followed by PBS (P1010, Solarbio, CN) washes. KNSTRN antibody (PA5-59828, Thermo Fisher, USA, 1:800 dilution) or CD8 antibody (RMA-0514, Fuzhou Maixin Biotech, CN, No dilution) was applied for 60 minutes, and after washing, the secondary antibody (HRP-conjugated secondary antibody (PV-6000, ZSGB-BIO, CN) was added for 15 minutes. DAB solution (PV-6000D, ZSGB-BIO, CN) was added for chromogenic detection and results observed within 10 minutes. Hematoxylin counterstaining followed, and differentiation was achieved in hydrochloric acid-ethanol before bluing under running water. Finally, slides were dehydrated in ethanol and xylene, and mounted using neutral gum and cover slips for preservation and analysis. The evaluation of KNSTRN utilized a histo-score (H-score) approach, which was determined by staining intensity and the proportion of positive cells. Staining intensity was categorized into four levels: 0 for none, 1 for weak, 2 for moderate, and 3 for strong. H-scores were computed using the following formula: H-score = [percentage of cells with intensity grade 1 (%)] + [percentage of cells with intensity grade 2 (%) × 2] + [percentage of cells with intensity grade 3 (%) × 3]. The density of positively CD8 stained cells was assessed by counting the stained cells observed in each field of view (cells/mm²).

### 2.15 Statistical analysis

The experiments noted above were carried out a minimum of three times. Statistical analysis was performed with GraphPad 8.0 and R (version 4.4.2), depicting continuous variables as the mean ± SD. The normality of variables was assessed utilizing the Shapiro-Wilk test prior to comparison. The Wilcoxon rank-sum test was utilized for comparing non-normally distributed variables between two groups. One-way ANOVA identified group differences attributable to a single treatment. The assessment of correlations between two continuous variables was performed using Spearman correlation coefficients (ρ). Statistical significance was determined with a two-tailed P value threshold of < 0.05.

## 3 Results

### 3.1 Expression of KNSTRN in different subtypes of breast cancer

**Figure 1:**
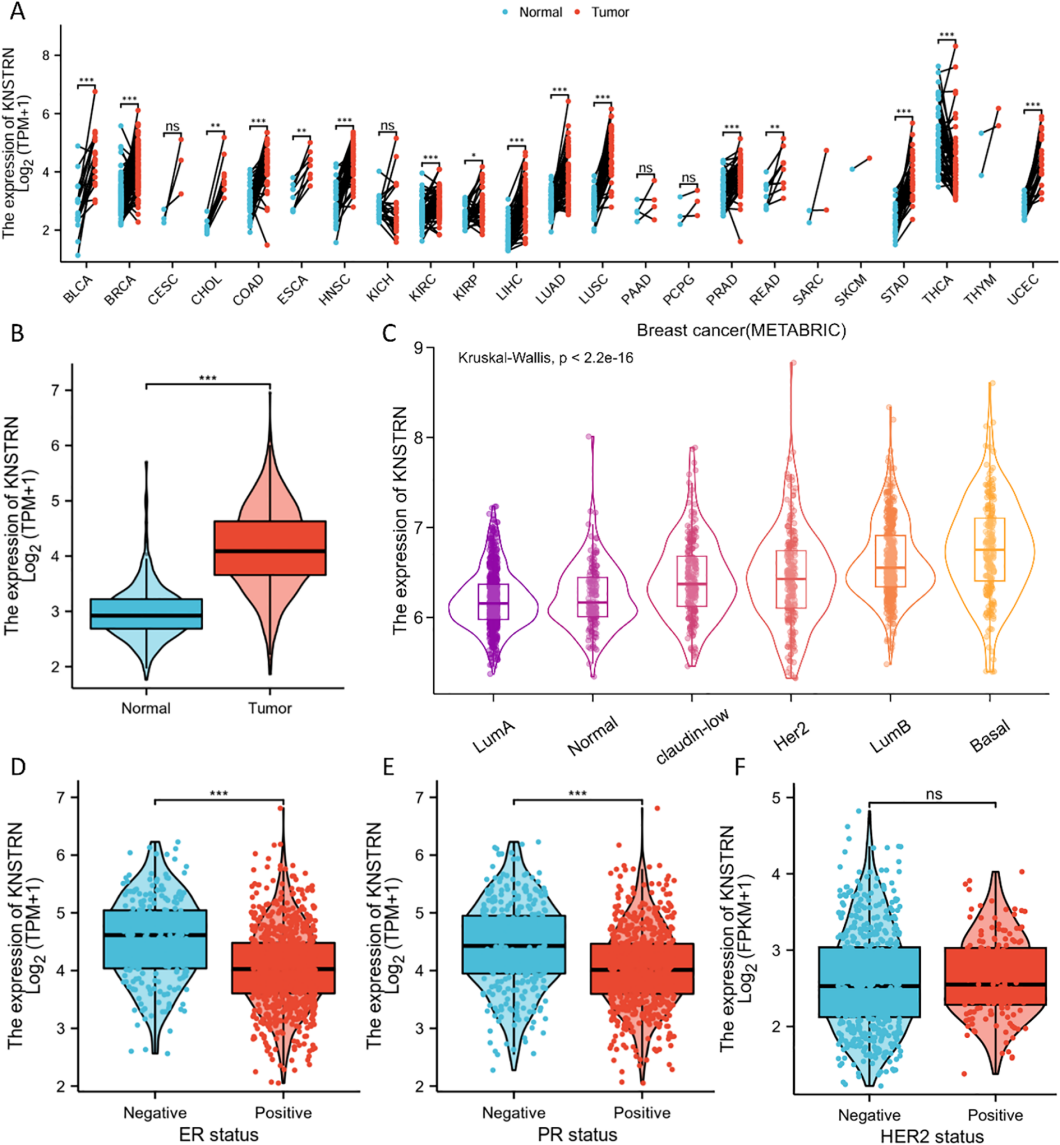
Expression of KNSTRN in different subtypes of breast cancer. **(A)** Expression of KNSTRN in different types of tumors. **(B)** Expression of KNSTRN in breast cancer and matched normal tissues. **(C)** Expression of KNSTRN in subtypes of PAM50 classification. **(D-F)** Associations between KNSTRN expression and clinicopathological characteristics including ER status, PR status and HER2 status.

Pan-cancer analysis revealed a pronounced increase in KNSTRN expression level across multiple malignancies in TCGA. Notably, elevated KNSTRN expression was observed in a range of cancers including breast cancer (BRCA) (**Figures 1A, B**). Furthermore, we analyzed the patient characteristics and expression data of KNSTRN to investigate its clinical significance. **Supplementary Table 1** provides detailed information on clinical features, which indicated that KNSTRN is significantly associated with clinical characteristics of breast cancer. We analyzed the expression levels of KNSTRN across different breast cancer subtypes classified by PAM50 in METABRIC and found that KNSTRN expression was higher in the basal-like subtype (**Figure 1C**). This subtype is also the predominant component of TNBC, with approximately 80% of basal subtype breast cancer being classified as TNBC (34). KNSTRN is elevated in ER-negative (**Figure 1D**) and PR-negative (**Figure 1E**) breast cancers. No significant differences in KNSTRN expression were observed between HER2-negative and HER2-positive subtypes (**Figure 1F**).

### 3.2 KNSTRN is highly expressed in TNBC and associated with prognosis

**Figure 2:**
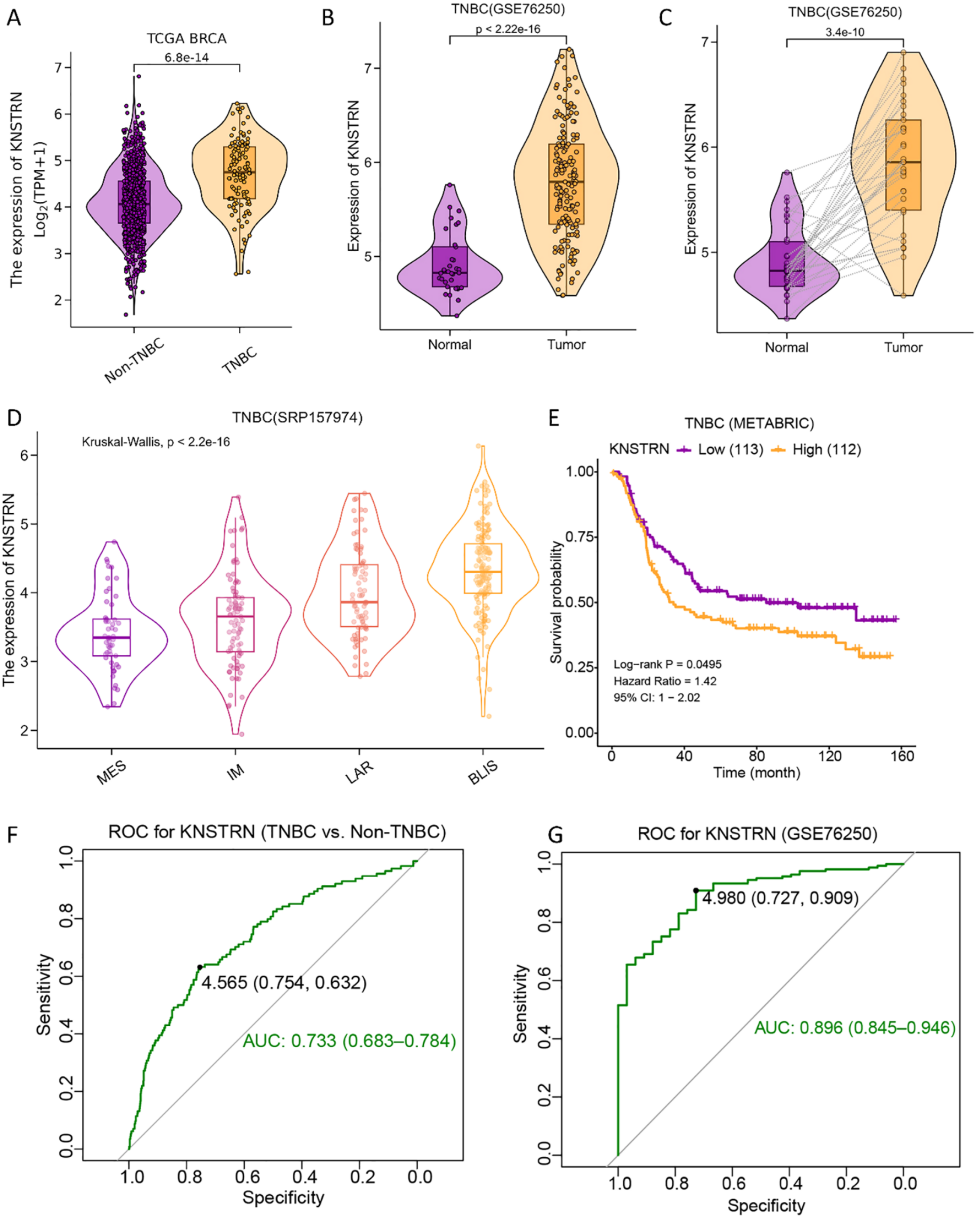
KNSTRN is highly expressed in TNBC and affects prognosis. **(A)** Expression of KNSTRN in TNBC and Non-TNBC of breast cancer. **(B)** Expression of KNSTRN in TNBC and non-matched normal tissues from GSE76250 dataset. **(C)** Expression of KNSTRN in TNBC and matched normal tissues from GSE76250 dataset. **(D)** Expression of KNSTRN in TNBC subtypes of FUSCC classification from SRP157974 dataset. **(E)** Relapse free survival (RFS) for TNBC patients with high versus low KNSTRN (data from METABRIC). **(F)** ROC curve for KNSTRN expression in differentiating TNBC from Non-TNBC (data from TCGA). **(G)** ROC curve for KNSTRN expression in TNBC (data from GSE76250).

**Figure 3:**
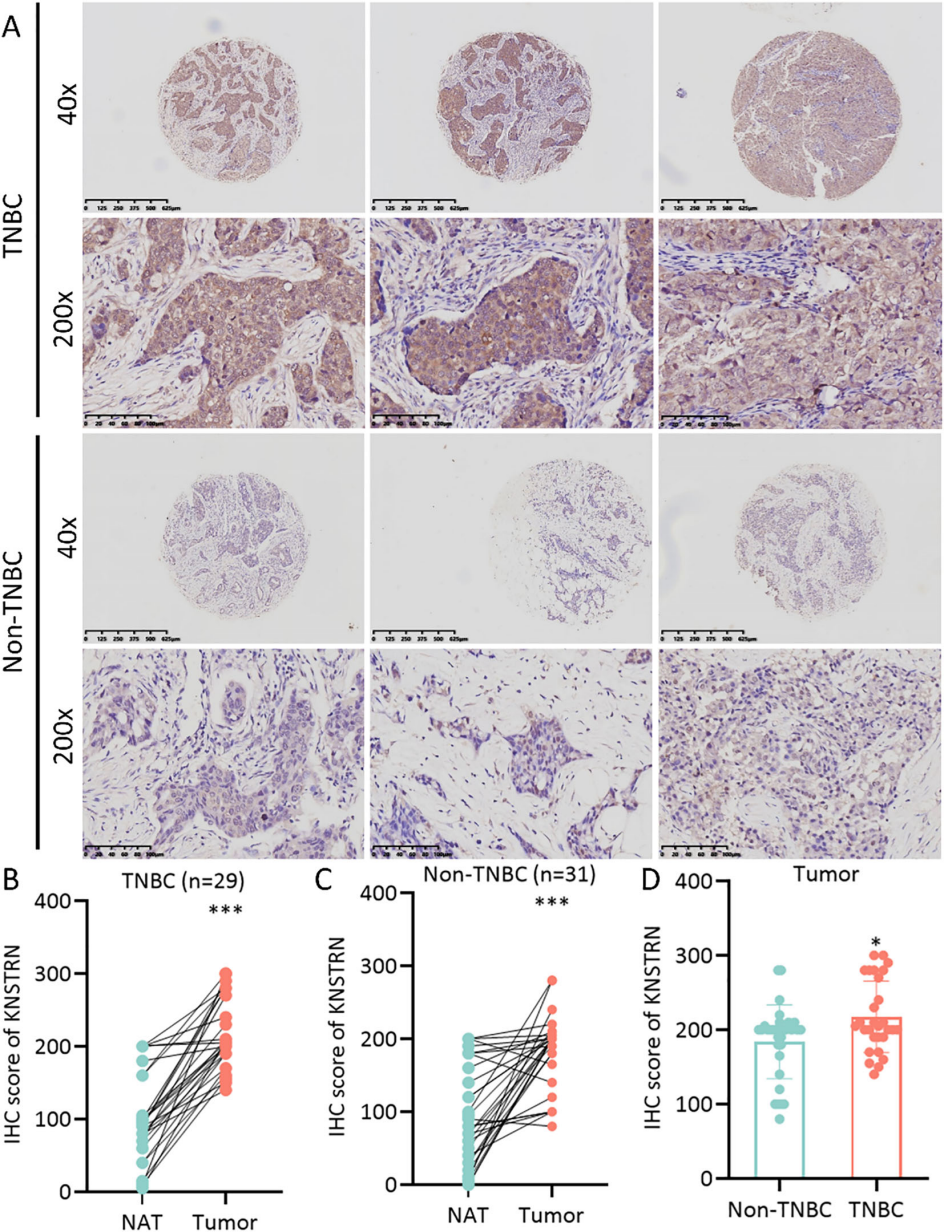
KNSTRN is highly expressed in TNBC patients. **(A)** The representative images of IHC staining of KNSTRN in breast cancer patients (TNBC and Non-TNBC). **(B)** The immunohistochemical scores of KNSTRN in TNBC patients. **(C)** The immunohistochemical scores of KNSTRN in Non-TNBC patients. **(D)** The Immunohistochemical Scores of KNSTRN in TNBC vs. Non-TNBC patients. NAT: Normal adjacent tissue. *P value < 0.05, ***P value < 0.001.

The expression level of KNSTRN in TNBC and Non-TNBC patients from TCGA database were analyzed. Our results showed that KNSTRN expression was significantly elevated in the TNBC group compared to the Non-TNBC group (**Figure 2A**). Subsequently, we validated the expression of KNSTRN in TNBC by analyzing GEO dataset (GSE76250), which revealed that KNSTRN was significantly overexpressed in cancerous tissues compared to normal tissues (**Figure 2B**) and cancerous tissues compared to matched adjacent normal tissues (**Figure 2C**). Moreover, the expression of KNSTRN in SRP157974 dataset varies among the different subtypes of TNBC according to the Fudan University Shanghai Cancer Center (FUSCC) classification (35), with the highest expression observed in the basal-like subtype (BLIS), which is associated with a higher degree of malignancy (**Figure 2D**). In addition, as for TNBC patients from METABRIC and SRP157974 datasets, those with elevated expression level of KNSTRN showed decreased recurrence free survival (RFS, **Figure 2E**, **Supplementary Figure 1**). To assess the diagnostic potential of KNSTRN expression, ROC curves were plotted. Our data indicated that KNSTRN’s expression levels possessed potential diagnostic capabilities of distinguishing TNBC from Non-TNBC (**Figure 2F**, AUC = 0.733, 95% CI: 0.683-0.784) and identifying TNBC from normal tissue (**Figure 2G**, AUC = 0.896, 95% CI: 0.845-0.946). The expression level of KNSTRN was calculated using log2(TPM + 1), with an expression threshold of 4.565 and 4.980 respectively. Based on this threshold, the accuracy of distinguishing TNBC patients from Non-TNBC patients is 73.3%, while the accuracy of identifying TNBC patients compared to healthy individuals is 89.6%. By performing immunohistochemical staining on patients’ samples to further validated KNSTRN expression levels in the tumor and NAT of patients with TNBC and Non-TNBC (**Figure 3A**), we observed that KNSTRN was expressed at higher levels in both TNBC and Non-TNBC tumor tissues compared to their NAT (**Figures 3B, C**). Additionally, KNSTRN expression was significantly higher in the tumor tissues of TNBC than in those of Non-TNBC patients (**Figure 3D**), which is consistent with our previously results.

### 3.3 Functional enrichment analysis of KNSTRN in TNBC

**Figure 4:**
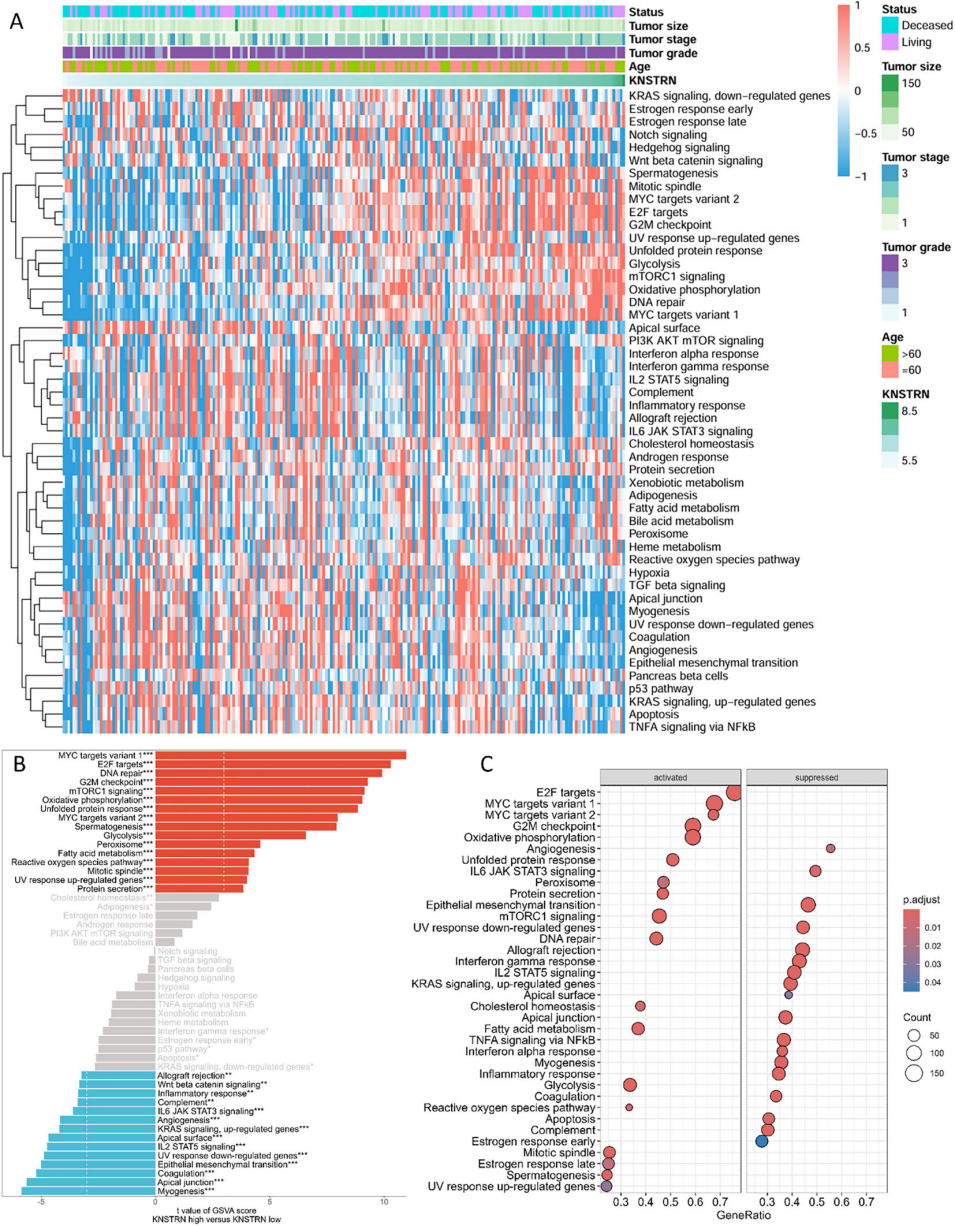
Functional enrichment analysis of KNSTRN-related DEGs in TNBC. **(A)** Heatmap for the correlation between KNSTRN and 50 hallmark signatures. **(B)** GSVA analysis of KNSTRN-related DEGs in TNBC. **(C)** GSEA analysis of KNSTRN-related DEGs in TNBC. *P value < 0.05; **P value < 0.01, ***P value < 0.001.

Patients with TNBC from the METABRIC cohort were categorized into two groups based on the expression levels of KNSTRN. Differential gene analysis revealed distinct gene expression profiles between the KNSTRN-low and KNSTRN-high groups. GSVA using hallmark gene sets from MSigDB demonstrated significant differences in the enrichment of various signaling pathways between these groups (**Figure 4**, **Supplementary Figure 2**). The enrichment score from the GSEA analysis is shown in **Supplementary Figure 3**. In the KNSTRN-low group, hallmark gene sets related to immune response such as IL2 STAT5 signaling, IL6 JAK STAT3 signaling, Inflammatory response, and Interferon gamma response were significantly enriched. Additionally, pathway associated with apoptosis (“Apoptosis”) were also highly enriched in this group. These findings suggest that KNSTRN-low TNBC may exhibit a stronger immune response and sensitivity to apoptosis. In contrast, the KNSTRN-high group exhibited significant enrichment of gene sets related to cell cycle regulation (“E2F targets”, “G2M checkpoint” and “Mitotic spindle”), metabolism (“Glycolysis”, “Oxidative phosphorylation”, Cholesterol homeostasis”, and “mTORC1 signaling”), and stress response (“Unfolded protein response”). Pathways associated with cell proliferation (“MYC targets variant 1/2”) and DNA repair (“DNA repair”) were also highly enriched in this group (P < 0.05). These results indicate that KNSTRN-high TNBCs may have enhanced proliferative capacity, metabolic activity, and resistance to stress. GO enrichment analysis was also performed and displayed in **Supplementary Figure 4**, which corroborates and extends the findings of the original GSEA.

### 3.4 KNSTRN expression in relation to the immunological landscape and tumor purity

**Figure 5:**
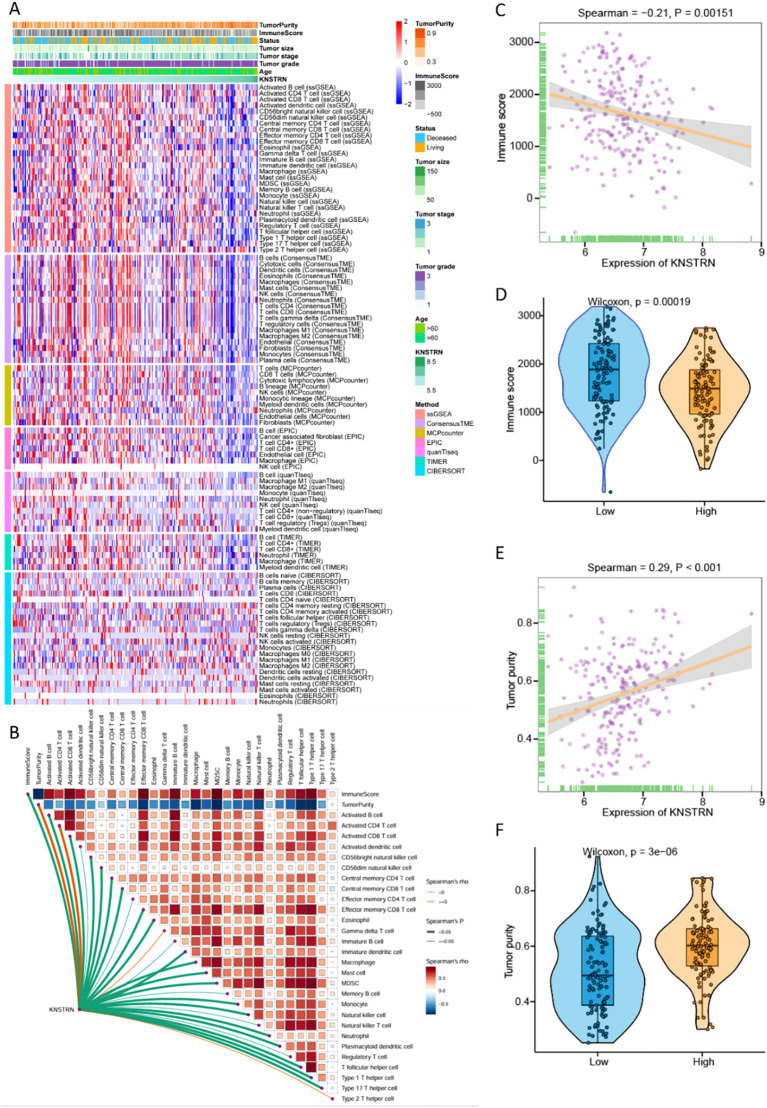
Relationship between KNSTRN expression and immune microenvironment of TNBC. **(A)** Heatmap for the correlation between KNSTRN and immune infiltration analysis based on CIBERSORT, ESTIMATE, MCPcounter, ssGSEA, and TIMER algorithms. **(B)** Correlations between KNSTRN and the relative abundance of immune cells. **(C)** Correlations between KNSTRN and immune score. **(D)** Comparisons of immune scores between the low-KNSTRN and high-KNSTRN groups. **(E)** Correlations between KNSTRN and tumor purity. **(F)** Comparisons of tumor purity between the low-KNSTRN and high-KNSTRN groups.

We comprehensively analyzed the relationship between KNSTRN expression and the immune microenvironment in TNBC using multiple bioinformatics algorithms and statistical methods. The heatmap (**Figure 5A**) illustrates the correlation between KNSTRN expression and immune infiltration based on. Our analysis revealed that KNSTRN expression is significantly negatively correlated with the relative abundance of immune cells. As indicated in **Figure 5B**, elevated KNSTRN is markedly associated with Activated B cells, Activated CD8+ T cells, Natural killer cell, Central memory CD4 T cell, Central memory CD8 T cell, Effector memory CD8 T cell, Eosinophil, Immature dendritic cell, Macrophage, Mast cell, MDSC, Monocyte, Natural killer cell, Neutrophil, Plasmacytoid dendritic cell, T follicular helper cell, Type 1 T helper cell and Type 17 T helper cell. Additionally, KNSTRN expression is significantly negatively correlated with the immune score (P = 0.0015; **Figure 5C**), indicating that higher KNSTRN expression is associated with a less immunogenic tumor microenvironment. Furthermore, KNSTRN expression is significantly positively correlated with tumor purity (P < 0.001; **Figure 5E**), suggesting that tumors with higher KNSTRN expression tend to have a lower proportion of immune cell infiltration. Comparisons of immune scores and tumor purity between low-KNSTRN and high-KNSTRN groups further confirmed these findings. The Wilcoxon rank-sum test showed that the immune score was significantly lower in the high-KNSTRN group compared to the low-KNSTRN group (**Figure 5D**), while tumor purity was significantly higher in the high-KNSTRN group (**Figure 5F**). These results collectively demonstrate that KNSTRN expression is associated with a less immunogenic and more tumor-pure microenvironment in TNBC.

**Figure 6:**
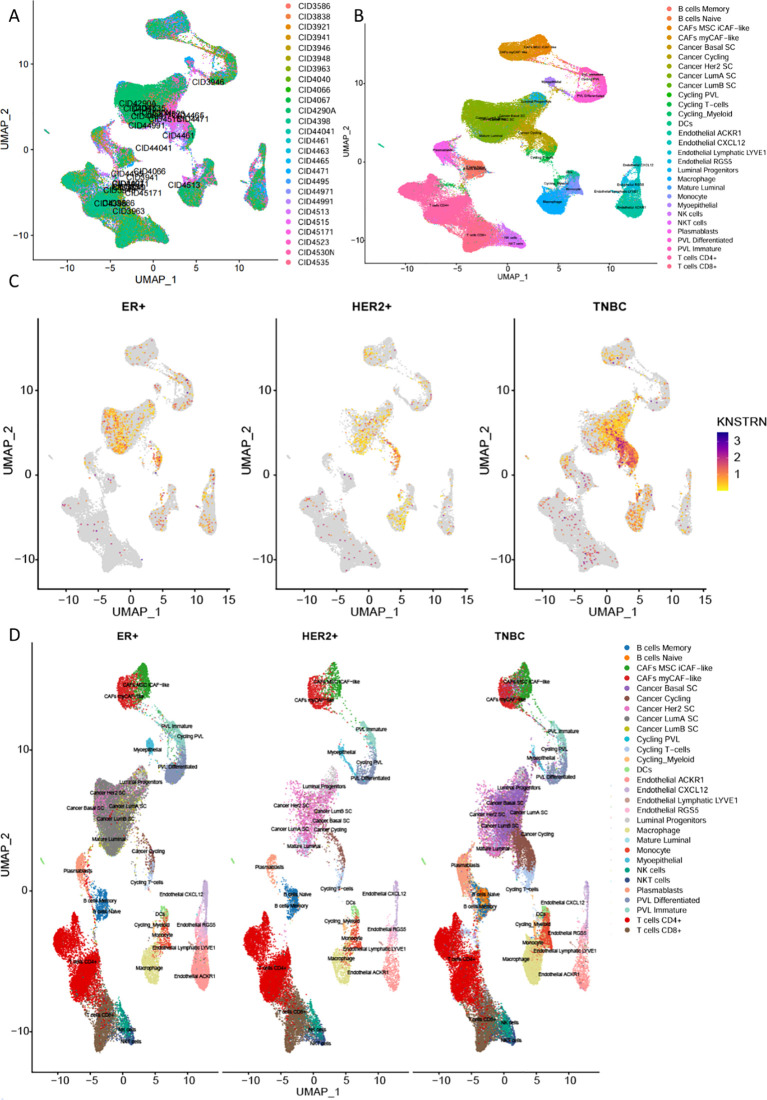
Expression of KNSTRN in single-cell RNA-Seq dataset. **(A)** Comparison of immune microenvironment cell types in different subtypes of breast cancer. **(B)** Cell annotation in the immune microenvironment of breast cancer. **(C)** KNSTRN expression in different subtypes of breast cancer. **(D)** UMAP analysis of the cell distribution among various breast cancer subtypes.

Subsequently, we conducted an analysis by using a single cell RNA-Seq dataset (GSE176078) (36). **Figure 6A** showed an integration of all samples with 26 primary tumors with 5 HER2+, 11 ER+ and 10 TNBCs cases, exhibiting a good integration without evident batch effects. The analysis revealed a total of 29 distinct cell types in breast cancer, with the top five most abundant being CD4+ and CD8+ T cells, Cancer LumA SC, Macrophages, as well as Cancer Cycling (**Figure 6B**, **Supplementary Figure 5**, **6**). Compared to the other two subtypes of breast cancer, TNBC exhibited significant high expression of KNSTRN (**Figure 6C**). Analysis of KNSTRN expression among various cell types in the three breast cancer subtypes reveals its upregulation in various immune cell types including CD8+ T cells, CD4+ T cells, macrophages, etc., and cancer-associated cells such as cancer cycling cells and cancer Her2 SC in TNBC (**Figure 6D**, **Supplementary Figure 7**). Analysis of the proportional KNSTRN-expression distribution among the major immune and malignant cell populations across the three breast cancer subtypes shows that KNSTRN is predominantly expressed in actively proliferating subsets such as Cycling Myeloid, Cancer cycling cells and Cycling PVL (**Supplementary Figure 8**).

### 3.5 KNSTRN in TNBC is associated with CD8+ T cell infiltration and immune-related genes

**Figure 7:**
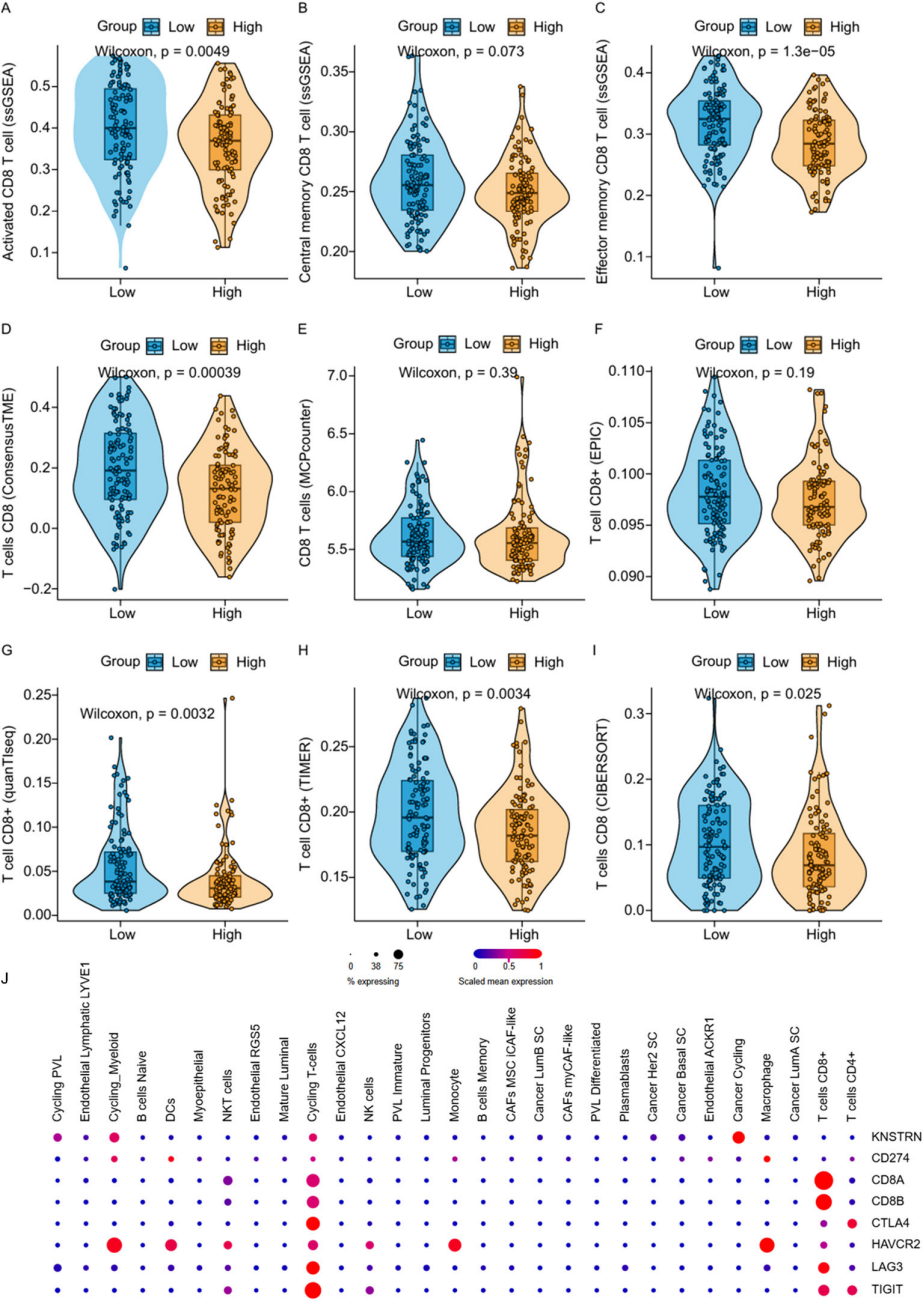
Correlation between KNSTRN expression and CD8+ T cell-associated signatures and immune-related genes. **(A–I)** Comparisons of CD8+ T cell-associated signatures between the low-KNSTRN and high-KNSTRN groups in TNBC. **(J)** Expression of KNSTRN and immune-related genes in single-cell RNA-seq dataset of TNBC (GSE176078). *P value < 0.05, **P value < 0.01.

**Figure 8:**
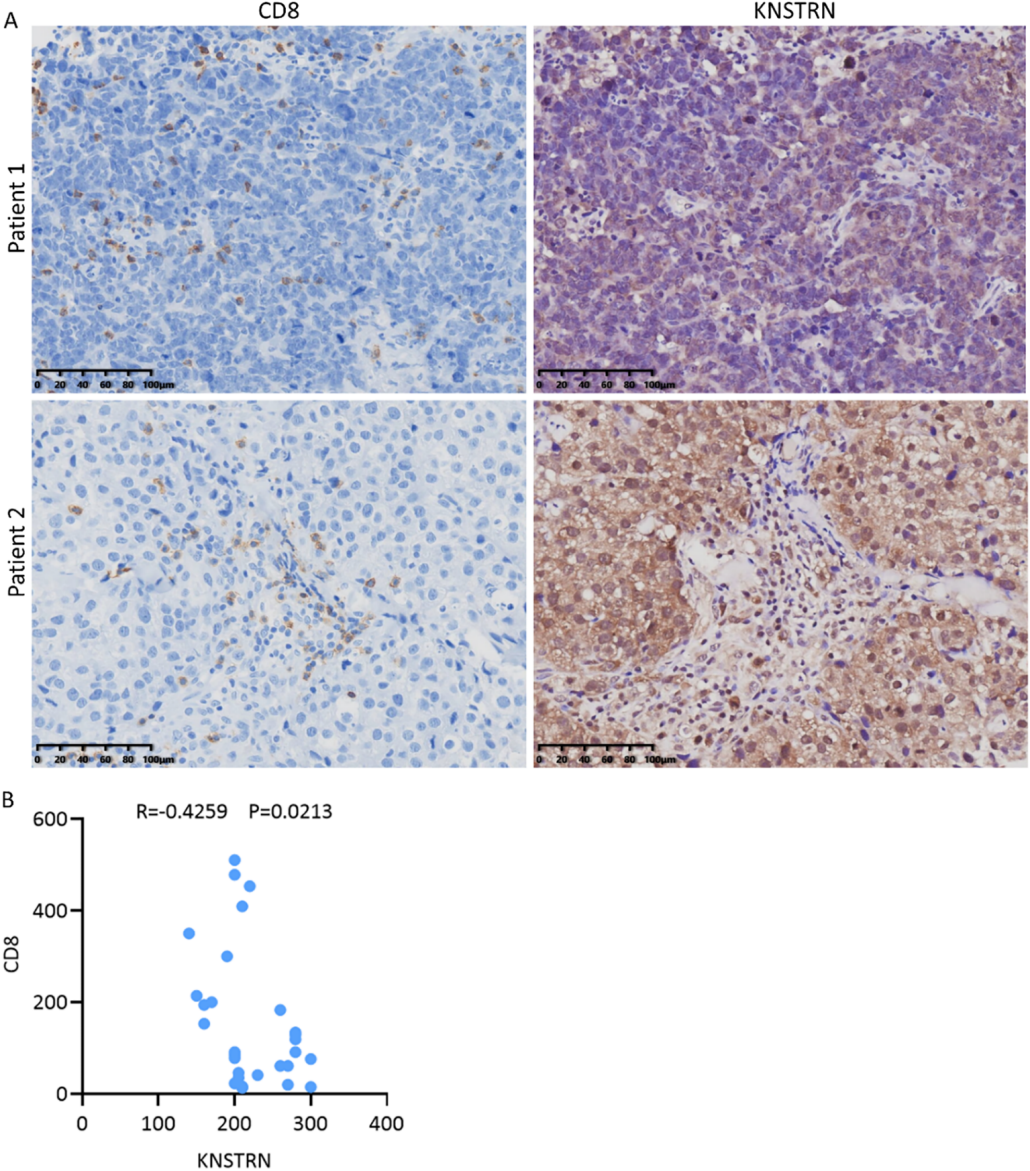
Reduced CD8+ T cell infiltration in TNBC patients with high expression of KNSTRN. **(A)** The representative images (200x) of IHC staining of KNSTRN and CD8 in TNBC patients (n=29). **(B)** Correlation analysis of KNSTRN and CD8 expression in TNBC patient specimens.

To explore the impact of KNSTRN on the immune landscape of TNBC, the immune infiltration data containing CD8+ T cells was extracted from **Figure 5A** for further analysis. By utilizing ssGSEA to analyze the infiltration of Activated CD8+ T cell, Central memory CD8+ T cell and Effector memory CD8+ T cell (**Figures 7A–C**) in the KNSTRN high-expression group and low-expression group, it was found that the abundance in the high-expression group was significantly lower than that in the low-expression group. We employed ConsensusTME, MCPcounter, EPIC, quanTIseq, TIMER, and CIBERSORT to analyze the infiltration of CD8+ T cells in TNBC patients with high and low expression of KNSTRN (**Figures 7D–I**). Except for MCPcounter and EPIC analyses, which did not show significant differences, the results from the other methods indicated that the abundance of CD8+ T cells in the high KNSTRN expression group was significantly lower than that in the low expression group. These findings suggest that CD8+ T cell abundance is significantly reduced in TNBC patients with high KNSTRN expression compared to those with low expression. Moreover, we employed ssGSEA to explore the relationship between KNSTRN expression and immune cell infiltration in TNBC. The results revealed that KNSTRN exhibited the strongest negative correlation with effector memory CD8+ T cells (**Supplementary Figure 9**). We validated this finding by using IHC in TNBC patients’ tissue and obtained the same negative correlation (**Figures 8A, B**). Next, we evaluated the association between KNSTRN and immune-related genes by using the single cell RNA-Seq dataset (GSE176078). In this dataset, we found that KNSTRN is primarily expressed in tumor cells. (**Figure 7J**). We systematically evaluated the correlations between KNSTRN expression and immune-related gene including the indicated immune checkpoint molecules in the METABRIC-TNBC dataset (**Supplementary Figure 10**). The data demonstrate that KNSTRN expression is significantly and negatively correlated with several key immune checkpoints such as PDCD1 and CTLA4, suggesting its potential association with poor prognosis and immune cell exclusion. To evaluate the predictive value of KNSTRN expression for immunotherapy response, we used the TIDE computational framework and CTR-DB 2.0 to assess the association between KNSTRN expression and response to immunotherapy. The results showed that the high-KNSTRN expression group exhibited a lower response rate to immunotherapy compared to the low-expression group, both methods show the consistent trends, although this difference did not reach statistical significance (**Supplementary Figure 11**).

### 3.6 Knockdown of KNSTRN inhibits the malignant characteristics of TNBC *in vitro*

**Figure 9:**
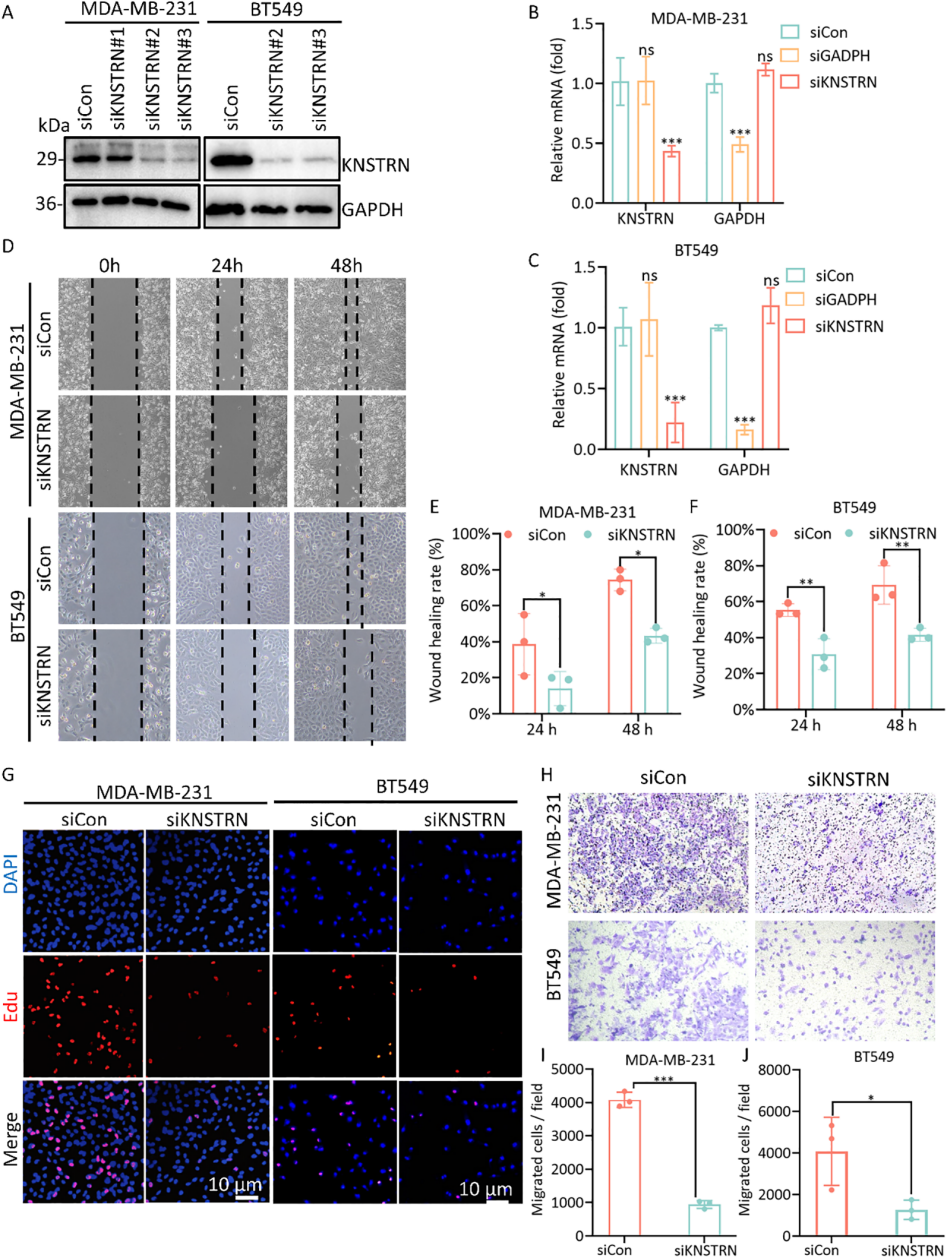
Knockdown of the expression of KNSTRN inhibits malignant characteristics of TNBC cells. **(A)** The transfection efficiency of siKNSTRN in the MDA-MB-231 and BT549 cell lines were detected by Western blotting. **(B, C)** The mRNA level in MDA-MB-231 and BT-549 cells transfected with siRNA. **(D)** Wound-healing assay showing delayed wound-healing of KNSTRN-downregulated MDA-MB-231 and BT549. **(E, F)** Wound healing rate of MDA-MB-231 and BT549. **(G)** EdU assay was applied to detect the efficiency of KNSTRN knockdown on the proliferation of MDA-MB-231 and BT549 cells. **(H)** Transwell assay was utilized to detect the changes in the migration ability of MDA-MB-231 and BT549 after KNSTRN silencing. **(I)** Migrated cells in siCon and siKNSTRN groups of MDA-MB-231. **(J)** Migrated cells in siCon and siKNSTRN groups of BT549. *P value < 0.05, ***P value < 0.001.

**Figure 10:**
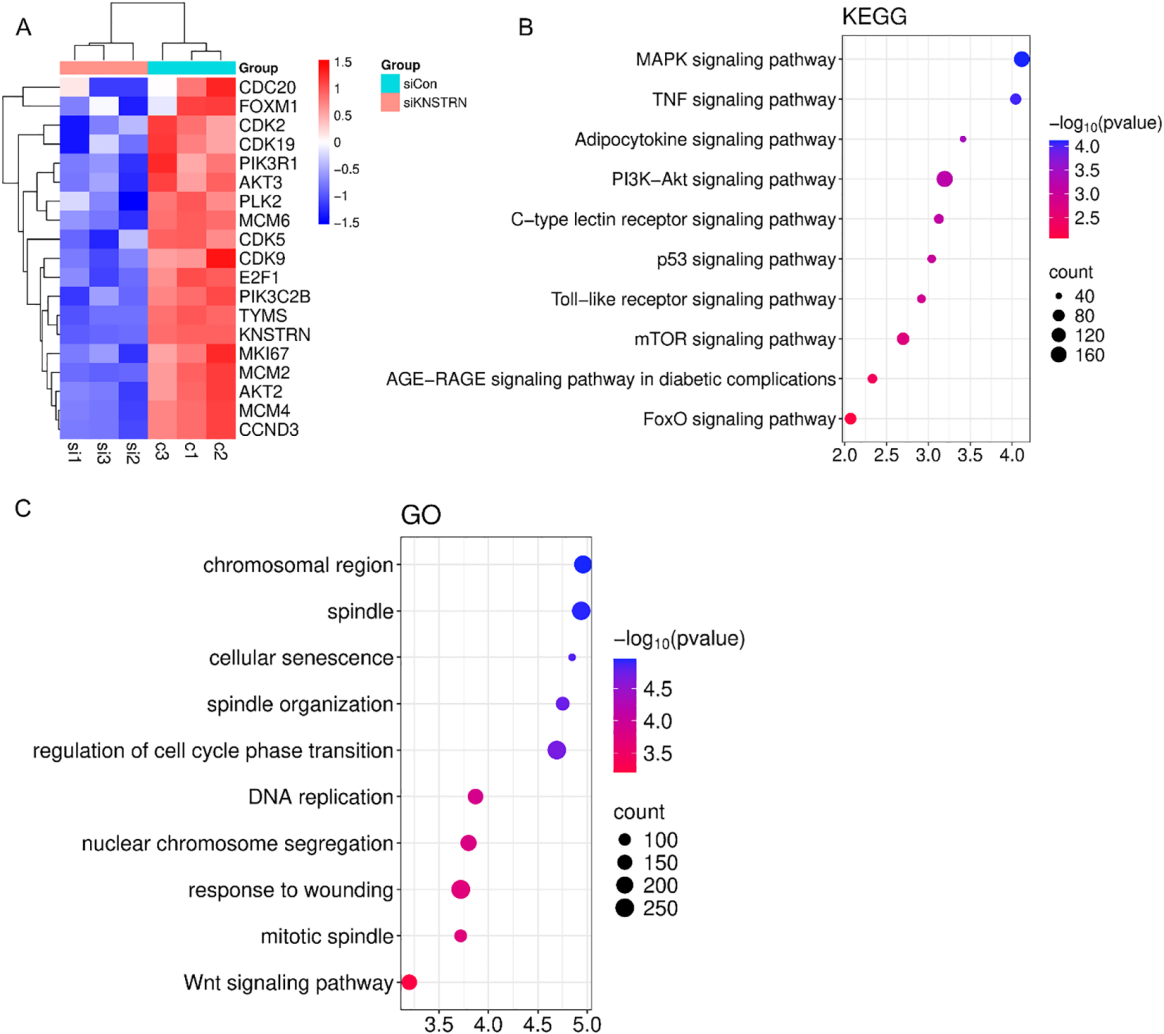
Transcriptomic profiling of BT549 cells after transfection of siKNSTRN or siControl. **(A)** Clustering plot of differentially expressed genes between the siKNSTRN and siControl groups. **(B)** KEGG pathway analysis of the significantly enriched pathways. **(C)** GO enrichment analysis of upregulated and downregulated DEGs.

Based on the previous results, it is evident that KNSTRN exhibits higher expression levels and exerts a greater impact in TNBC of higher malignancy. As a result, we opted to explore the impact of KNSTRN in TNBC using the MDA-MB-231 and BT549 cell line for transfection of siRNA targeting KNSTRN. The knockdown efficiency of siKNSTRN and siGAPDH was verified by Western blot (**Figure 9A**, **Supplementary Figure 12**). Among the siRNAs tested, siKNSTRN#3 had better silencing efficacy and it was used for subsequent experiments. The knockdown efficiency of siKNSTRN was verified by qRT–PCR as well (**Figures 9B, C**). We assessed the impact of KNSTRN on the ability to migrate of TNBC cells. Wound healing (**Figures 9D–F**) and transwell assays (**Figures 9H–J**) revealed a noteworthy reduction in the migratory potential of cells after siKNSTRN. EdU, a thymidine analog, is capable of substituting thymidine in the process of DNA replication and integrating into the elongating DNA strand. Utilizing the distinct interaction between Apollo^®^ fluorescent dye and EdU enables the straightforward and exact observation of DNA synthesis activity. Consistently, experiments employing EdU revealed a significant decrease in DNA synthesis activity within cells belonging to the KNSTRN-knockdown group in contrast to those in the control group (**Figure 9G**). In addition, we conducted RNA sequencing on BT549 cells following KNSTRN knockdown (**Figure 10**). Applying a threshold of |log2FoldChange| ≥ 1 and p-value < 0.05, we identified a total of 397 up-regulated and 1141 down-regulated DEGs. KNSTRN was significantly knocked down in siKNSTRN group compared to siControl group (**Figure 10A**). The clustering analysis of DEGs between the siKNSTRN group and the siControl group reveals that KNSTRN knockdown leads to reduced expression levels of key cell cycle regulators (e.g., CDK2, CDC20, FOXM1, E2F1), essential DNA replication genes (MKI67, MCM2, MCM6), as well as critical components of the PI3K-AKT signaling pathway associated with cell proliferation (PIK3R1, PIK3C2B, AKT2, AKT3). The transcriptomic data also revealed that silencing of the KNSTRN gene affected pathways related to cell proliferation and mitosis (e.g., mTOR signaling pathway, mitotic spindle) (**Figures 10B, C**), which is consistent with the functional enrichment results obtained from our bioinformatic analysis.

## 4 Discussion

KNSTRN is a critical protein involved in mitotic spindle function and accurate chromosome segregation. Elevated KNSTRN expression is linked to poor prognosis and altered immune infiltration in pan-cancers (18). It associates with increased immunosuppressive cells and reduced cytotoxic CD8^+^ T and NK cells in pan-cancer research. In lung and hepatocellular carcinomas, KNSTRN correlates with Th2 polarization and T-cell exhaustion markers (15, 16). KNSTRN also contributes to therapy resistance potentially through ER stress pathways in bladder cancer (18, 19), and its genetic variants underscore its potential as a diagnostic and therapeutic target (20).

In our research, we systematically investigated the functional role of KNSTRN in TNBC progression. We found that KNSTRN is significantly upregulated in TNBC compared to Non-TNBC and normal breast tissues, correlating with more aggressive subtype of TNBC and poorer recurrence free survival. ROC curves indicated that KNSTRN’s expression levels possessed potential diagnostic capabilities of distinguishing TNBC from Non-TNBC (AUC = 0.733, 95% CI: 0.683-0.784) and identifying TNBC from normal tissue (AUC = 0.896, 95% CI: 0.845-0.946). This suggested that KNSTRN was a candidate biomarker for TNBC diagnosis and prognosis. Transcriptomic analysis of the METABRIC TNBC cohort revealed that KNSTRN-high tumors are enriched for signatures of active cell cycling, including E2F targets, MYC targets, and G2/M checkpoint signaling, and PI3K-AKT-mTOR signaling, etc. This finding suggests that KNSTRN may promote TNBC progression through cell cycle regulation and proliferative signaling. This transcriptional profile was functionally validated through *in vitro* experiments. siRNA-mediated knockdown of KNSTRN in MDA-MB-231 and BT549 cells resulted in a significant reduction in EdU incorporation, demonstrating suppressed DNA replication and decreased cellular proliferation. Wound healing and Transwell assays showed that knockdown of KNSTRN in cell lines led to slowed migration, indicating that KNSTRN can promote cancer cell progression. Further, RNA-seq of BT549 cells after siKNSTRN revealed pronounced downregulation of critical cell cycle-related genes, including CDK2, CDC20, FOXM1, and E2F1, which have been documented to drive uncontrolled proliferation and tumor growth in TNBC (37–40). Key components of the PI3K-AKT pathway including PIK3R1, PIK3C2B and AKT2 and AKT3were also significantly suppressed. Additionally, PI3K-AKT signaling and mTORC1 signaling were enriched in KEGG enrichment analysis. This axis is a well-established critical regulator of cell proliferation in TNBC, and its hyperactivation is a known oncogenic driver associated with poor prognosis (41). Collectively, these results lead us to propose that KNSTRN facilitates TNBC progression potentially by driving cell cycle progression and activating the PI3K-AKT-mTOR signaling axis, thereby promoting cell proliferation and tumor growth.

KNSTRN expression is also closely linked to the tumor immune microenvironment. Our analysis showed that there was significantly negative correlation between high KNSTRN expression and most of the immune cell infiltration, particularly CD8+ T cells. And ssGSEA analysis revealed that the most significant negative correlation between KNSTRN and effector memory CD8+ T cells in TNBC. This finding is significant because that CD8+ T cells are not only predictive of outcomes in breast cancer patients treated with immune checkpoint inhibitors but also serve as critical indicators for monitoring the efficacy of neoadjuvant therapy (42). Moreover, CD8 is used as a marker in the FUSCC classification to predict the clinical outcomes and guide treatment decisions for patients with immune-modulatory subtype of TNBC (35). Single-cell data indicated that CD8+ T cells exhibited high expression of LAG3 and TIGIT, which suggested that these cells may be in a state of exhaustion (18, 43, 44). The high expression of HAVCR2 (TIM-3) in macrophages further supported the existence of an immunosuppressive milieu that underlies this T cell exhaustion. These molecules play a key role in immune suppression within the TNBC microenvironment. Upon engagement with their respective ligands, these receptors inhibit T cell proliferation and effector functions, thereby facilitating tumor immune evasion (45). These indicated KNSTRN contributed to an exhausted immune microenvironment, facilitating immune escape. And it can be inferred that KNSTRN may contribute to CD8^+^ T cell dysfunction through several interrelated mechanisms. Transcriptomic profiling reveals that KNSTRN-high tumors exhibit metabolic alteration within the tumor microenvironment, which potentially creating a nutrient-depleted and acidic microenvironment that can suppress T cell function. Additionally, KNSTRN expression positively correlates with tumor purity, suggesting a possible reduction in stromal and immune cell infiltration that may limit proper CD8^+^ T cell activation. These findings indicated that KNSTRN may induce an exhausted state in CD8^+^ T cells by promoting tumor cell proliferation, altering tumor metabolism, and CD8^+^ T cell exhaustion.

Other cells that show obvious correlation with KNSTRN expression levels including natural killer cell, macrophages, T helper cell etc. This suggests that KNSTRN may contribute to immune evasion in TNBC by suppressing the infiltration and activity of immune cells. Although KNSTRN expression shows a positive correlation with activated CD4 T cells, its significant negative association with specific immunostimulatory subsets (Th1, Th17, and T follicular helper cells) of CD4+ T cell suggests a skewing of the immune response towards tolerance and suppression (46), which is consistent with its overall immunosuppressive role in the TNBC microenvironment. Moreover, elevated expression levels of KNSTRN are negatively correlated with immune score and tumor purity, further indicating that KNSTRN may contribute to immune suppression and a tumor-dominant microenvironment.

Our analysis based on TIDE and CTR-DB 2.0 revealed a non-significant but consistent trend suggesting that high KNSTRN expression is associated with reduced immunotherapy response. While this trend was observed across two independent computational platforms, the findings did not reach statistical significance, highlighting the need for further validation in larger and prospective clinical cohorts to more definitively assess the potential role of KNSTRN as a predictive biomarker.

There are some limitations in our study. The findings are primarily based on bioinformatics analysis and *in vitro* experiments, and further validation based on *in vivo* experiments is needed. The mechanisms by which KNSTRN regulates immune infiltration and tumor progression remain unclear, and its negative correlation with immune cells may involve factors like cytokine changes, metabolic alterations, and hypoxia (34, 47, 48).

## 5 Conclusions

In summary, our study provides evidences that KNSTRN is a candidate biomarker for TNBC prognosis and a potential target for therapeutic strategies. Its overexpression in TNBC is associated with aggressive tumor behavior and an immunosuppressive microenvironment, highlighting its significance in TNBC pathogenesis and prognosis. Future studies should focus on validating these findings in larger cohorts and exploring the potential mechanism in TNBC.

## Data Availability

The original contributions presented in the study are included in the article/**Supplementary Material**. Further inquiries can be directed to the corresponding authors.
