# Supplementary material for: Elevated KNSTRN as a potential indicator for triple-negative breast cancer progression and immune infiltration
Source: Front Immunol. 2025 Oct 23;16:1572359. doi: 10.3389/fimmu.2025.1572359 (PMC12589078; doi:10.3389/fimmu.2025.1572359)
Supplement: Supplementary file 1 [file Table1.docx]

Supplementary Material

# Supplementary Table

**Supplementary Table 1**. Clinical characteristics of high-KNSTRN and low-KNSTRN expression groups.

| Characteristics | Low expression of KNSTRN | High expression of KNSTRN | P |
| --- | --- | --- | --- |
| n | 543 | 544 |  |
| Age, n (%) |  |  | < 0.001 |
| <= 60 | 272 (25%) | 331 (30.5%) |  |
| > 60 | 271 (24.9%) | 213 (19.6%) |  |
| Pathologic T stage, n (%) |  |  | < 0.001 |
| T1 | 175 (16.1%) | 103 (9.5%) |  |
| T2 | 278 (25.6%) | 353 (32.6%) |  |
| T3 | 76 (7%) | 64 (5.9%) |  |
| T4 | 14 (1.3%) | 21 (1.9%) |  |
| Pathologic stage, n (%) |  |  | 0.005 |
| Stage I | 113 (10.6%) | 69 (6.5%) |  |
| Stage II | 291 (27.4%) | 328 (30.9%) |  |
| Stage III | 122 (11.5%) | 122 (11.5%) |  |
| Stage IV | 8 (0.8%) | 10 (0.9%) |  |
| Race, n (%) |  |  | 0.001 |
| Asian | 20 (2%) | 40 (4%) |  |
| Black or African American | 79 (7.9%) | 103 (10.3%) |  |
| White | 405 (40.6%) | 350 (35.1%) |  |
| Histological type, n (%) |  |  | < 0.001 |
| Infiltrating Ductal Carcinoma | 326 (33.2%) | 450 (45.9%) |  |
| Infiltrating Lobular Carcinoma | 157 (16%) | 48 (4.9%) |  |
| PR status, n (%) |  |  | < 0.001 |
| Negative | 122 (11.8%) | 220 (21.2%) |  |
| Indeterminate | 2 (0.2%) | 2 (0.2%) |  |
| Positive | 395 (38.1%) | 297 (28.6%) |  |
| ER status, n (%) |  |  | < 0.001 |
| Negative | 67 (6.4%) | 173 (16.7%) |  |
| Indeterminate | 0 (0%) | 2 (0.2%) |  |
| Positive | 452 (43.5%) | 345 (33.2%) |  |
| PAM50, n (%) |  |  | < 0.001 |
| Normal | 31 (2.9%) | 9 (0.8%) |  |
| LumA | 406 (37.4%) | 158 (14.5%) |  |
| LumB | 39 (3.6%) | 167 (15.4%) |  |
| Her2 | 26 (2.4%) | 56 (5.2%) |  |
| Basal | 41 (3.8%) | 154 (14.2%) |  |
| Menopause status, n (%) |  |  | 0.032 |
| Pre | 103 (10.6%) | 127 (13%) |  |
| Peri | 16 (1.6%) | 24 (2.5%) |  |
| Post | 376 (38.5%) | 330 (33.8%) |  |
| Anatomic neoplasm subdivisions, n (%) |  |  | 0.006 |
| Left | 260 (23.9%) | 306 (28.2%) |  |
| Right | 283 (26%) | 238 (21.9%) |  |
| OS event, n (%) |  |  | 0.037 |
| Alive | 479 (44.1%) | 456 (42%) |  |
| Dead | 64 (5.9%) | 88 (8.1%) |  |

# Supplementary Figure


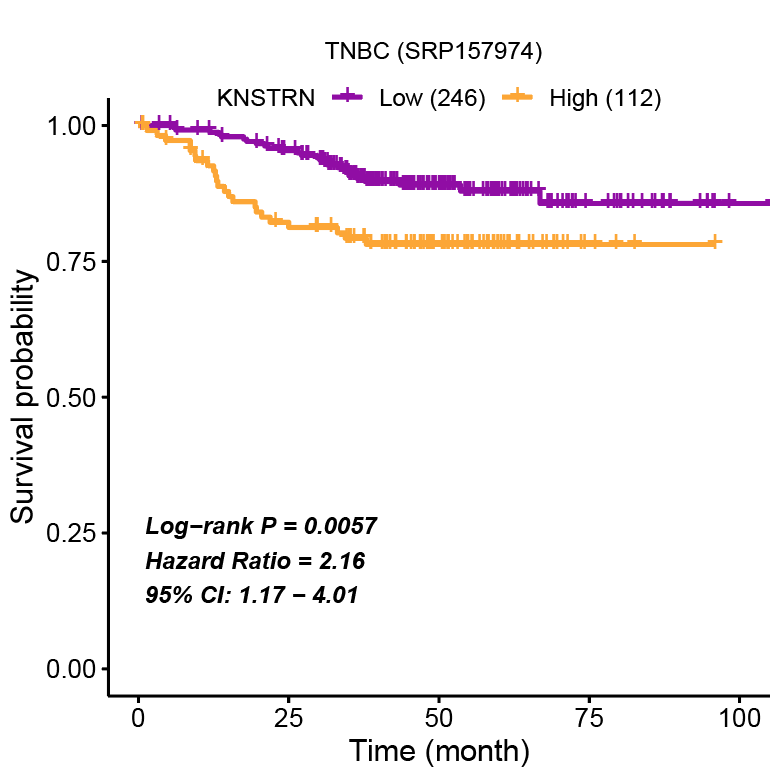


**Supplementary Figure 1**. Relapse free survival for TNBC patients with high versus low KNSTRN (data from SRP157974 dataset).


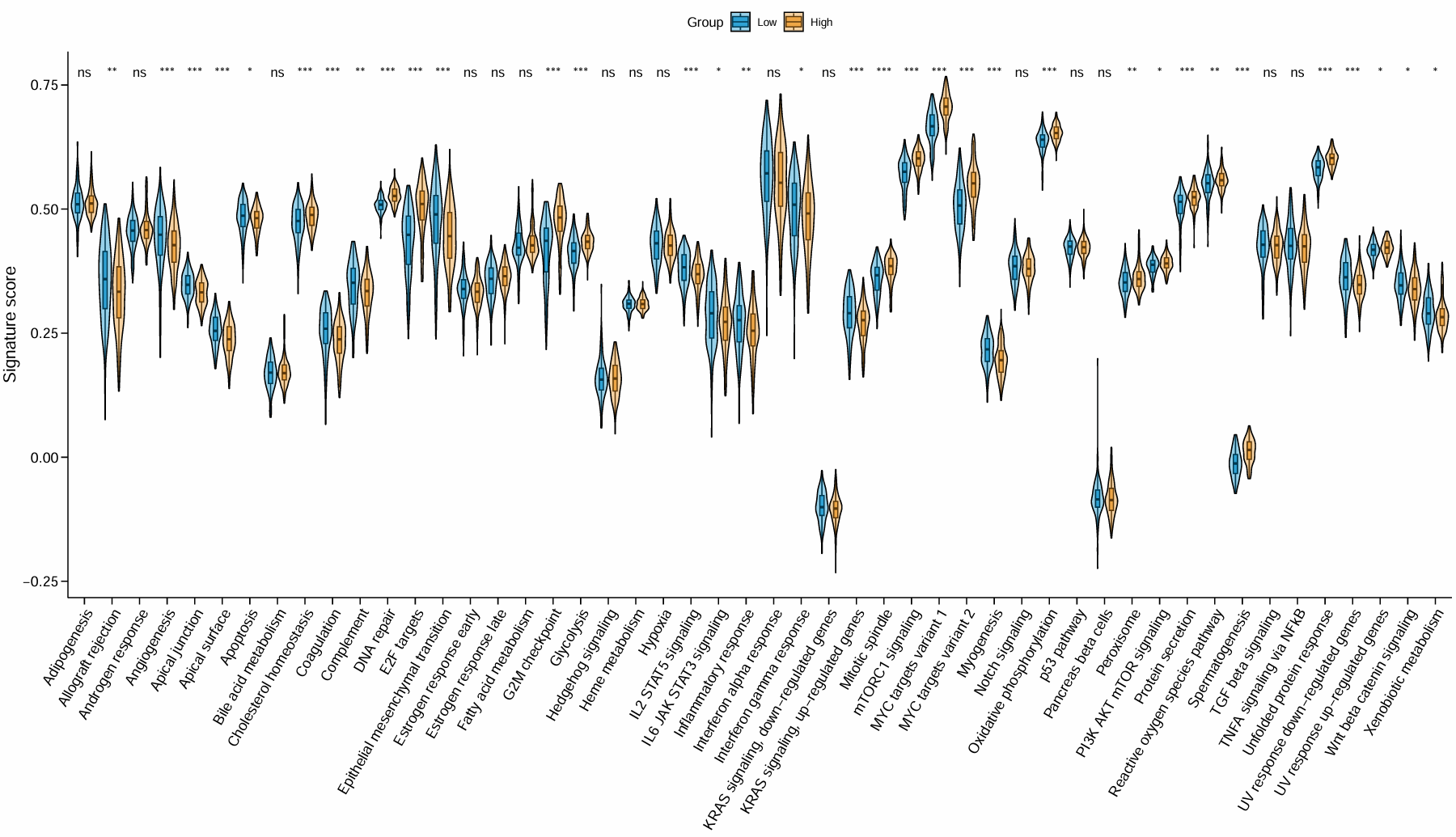


**Supplementary Figure 2**. Boxplot for the correlation between KNSTRN and 50 hallmark signatures.


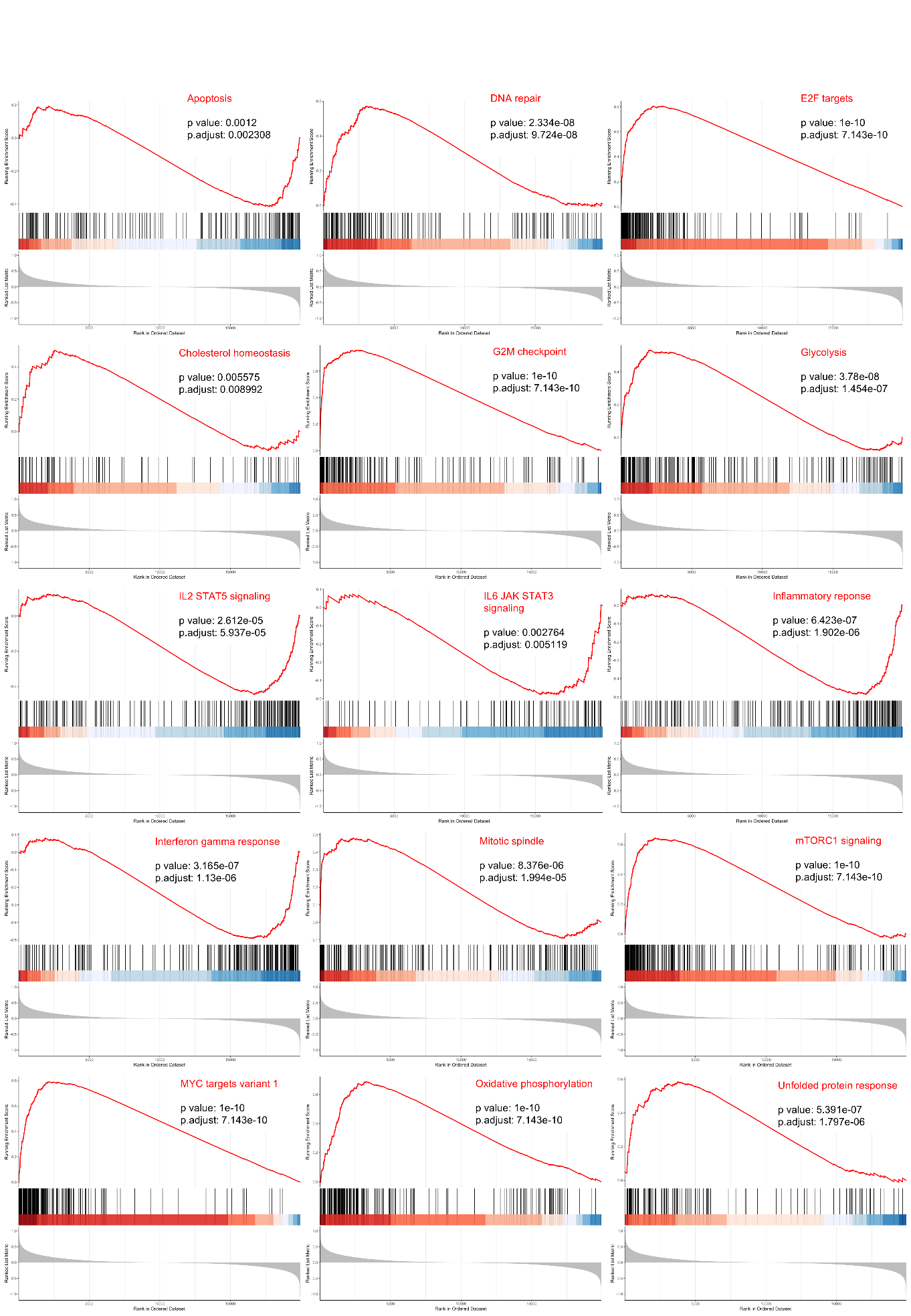


**Supplementary Figure 3**. Enrichment score plot for GSEA analysis of KNSTRN-related DEGs in TNBC from METABRIC dataset.


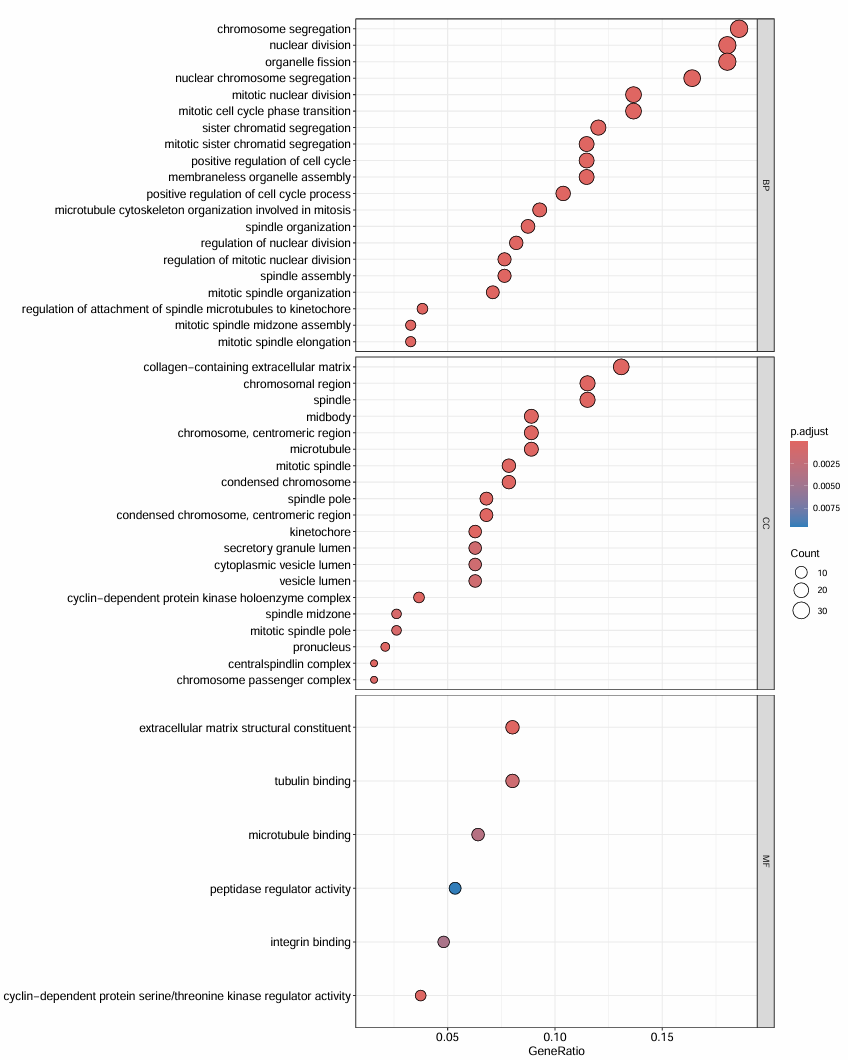


**Supplementary Figure 4**. GO analysis of KNSTRN-related DEGs in TNBC from METABRIC dataset.


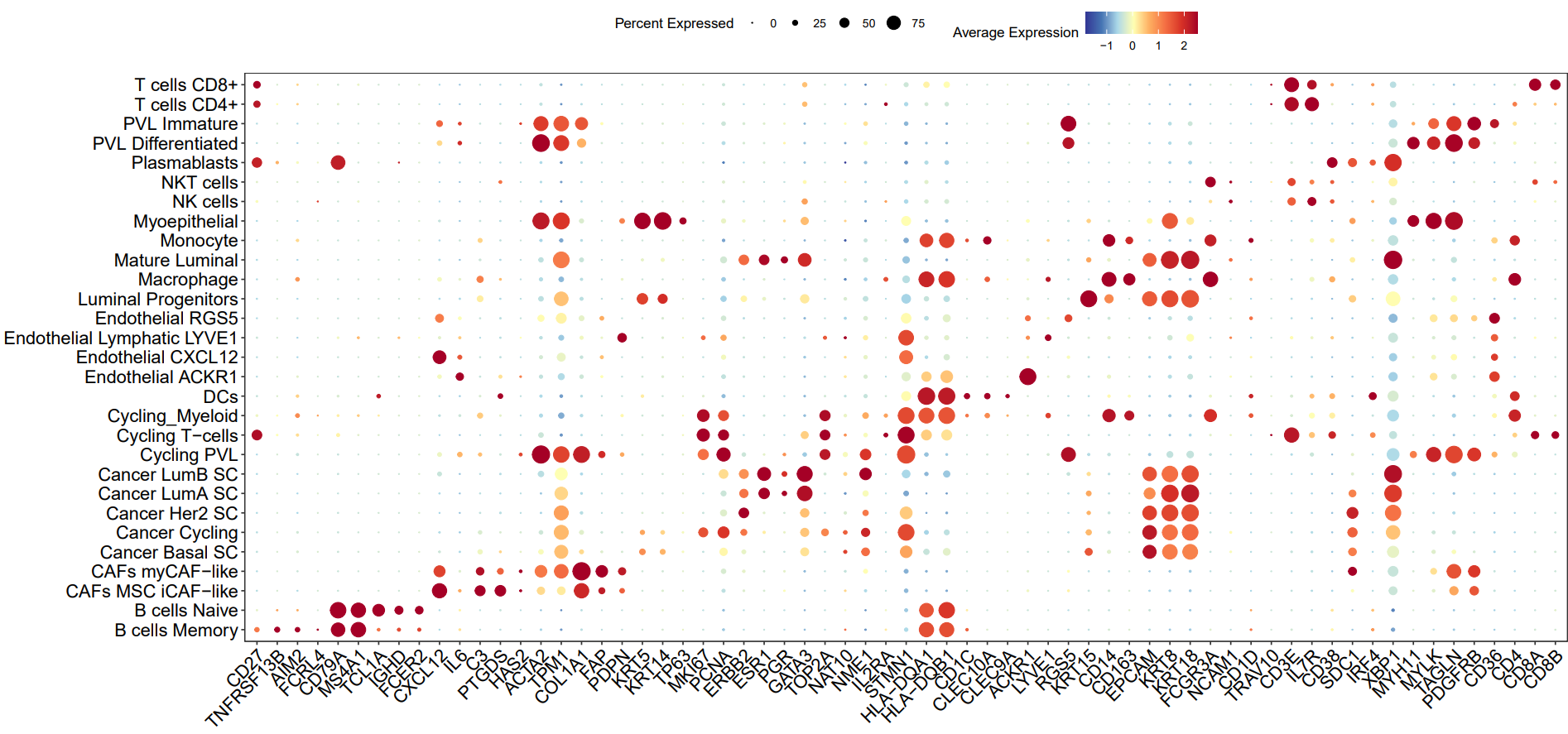


**Supplementary Figure 5**. Cell type annotations using canonical marker expression through DotPlot (data from GSE176078).


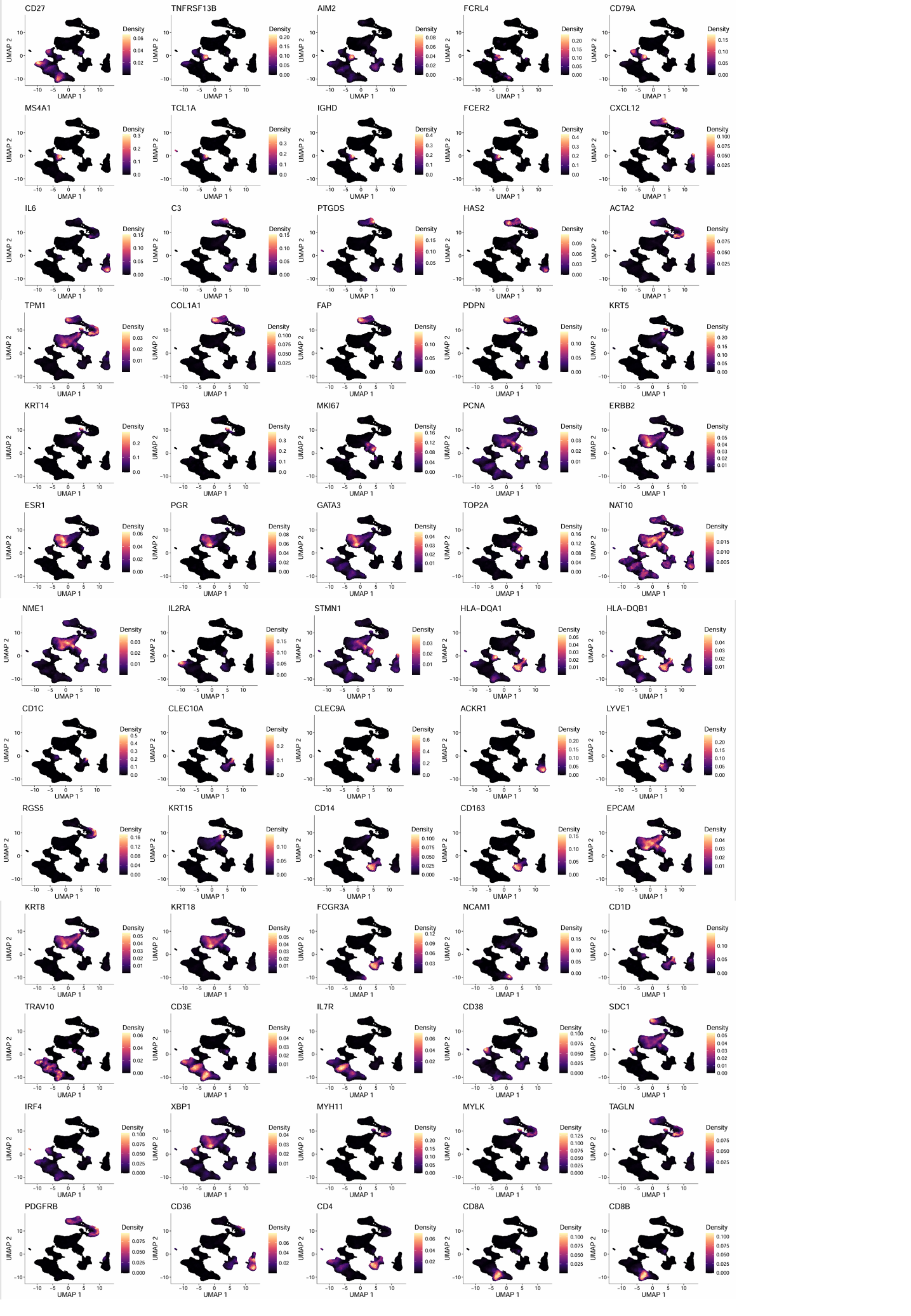


**Supplementary Figure 6.** Cell type annotations using canonical marker expression through FeaturePlot (data from GSE176078).


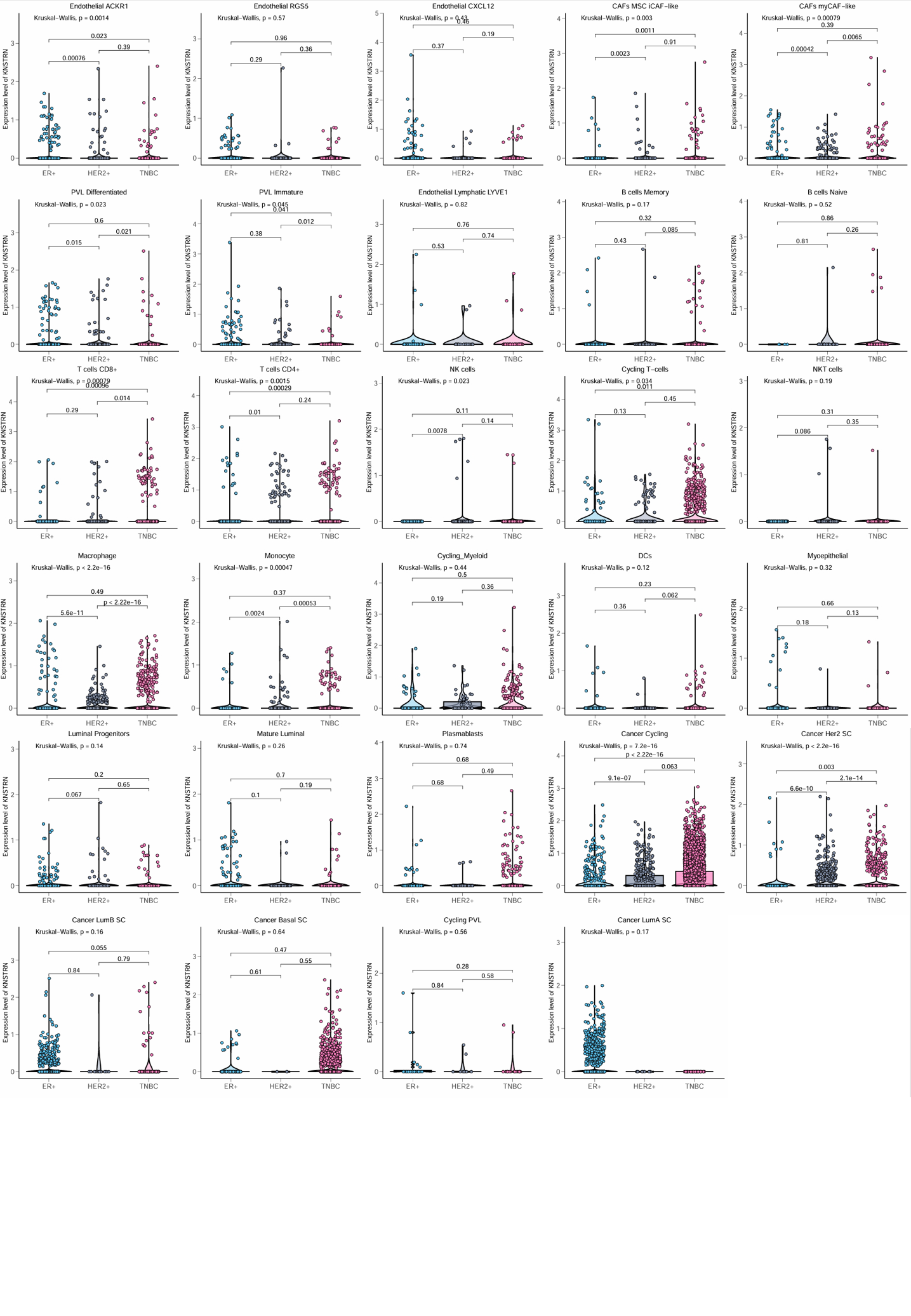


**Supplementary Figure 7.** Differential KNSTRN expression across tumor-microenvironment cell subpopulations among breast cancer subtypes (data from GSE176078).


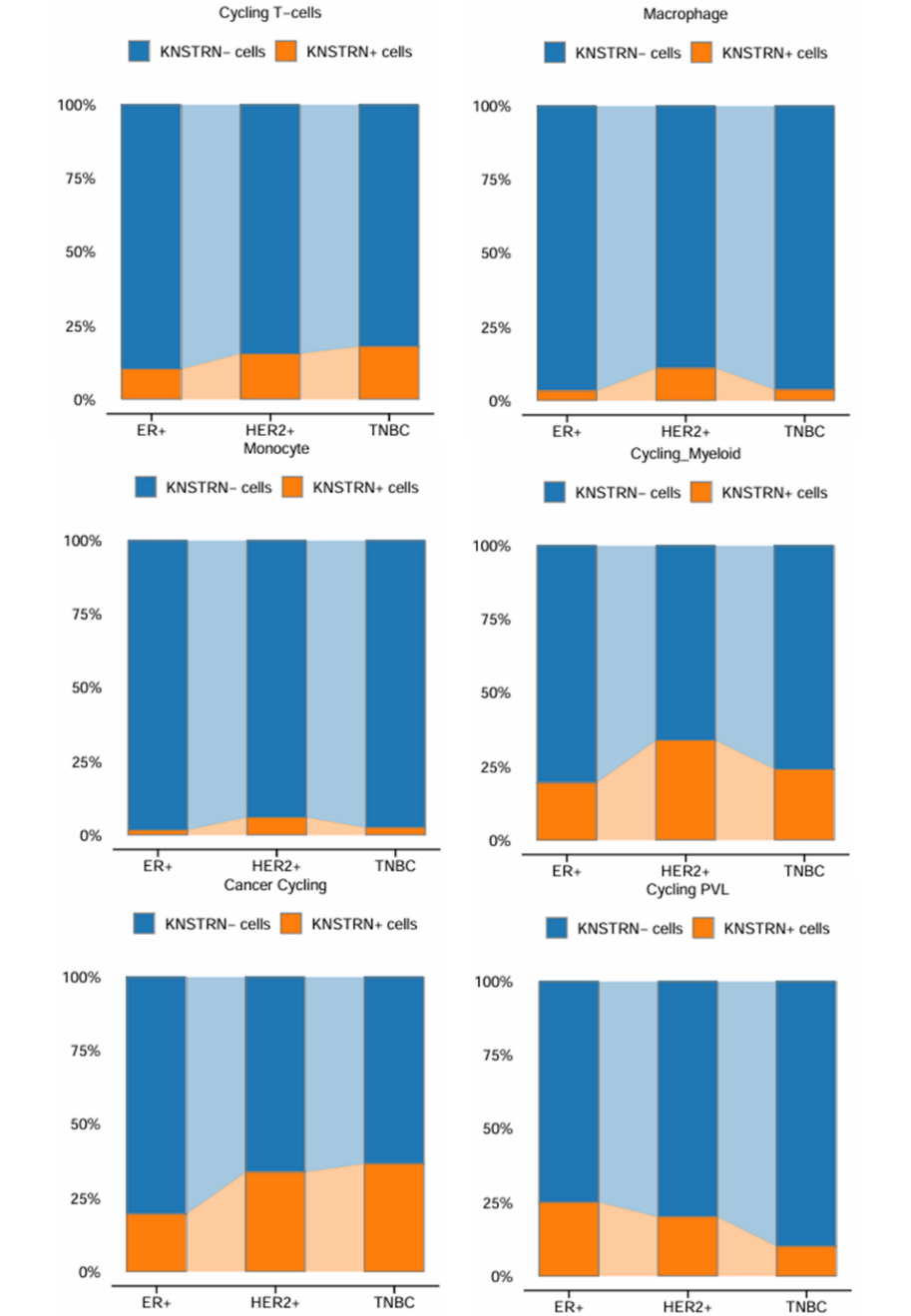


**Supplementary Figure 8**. Relative proportion of major immune and tumor cell populations across the three subtypes of breast cancer (data from GSE176078).


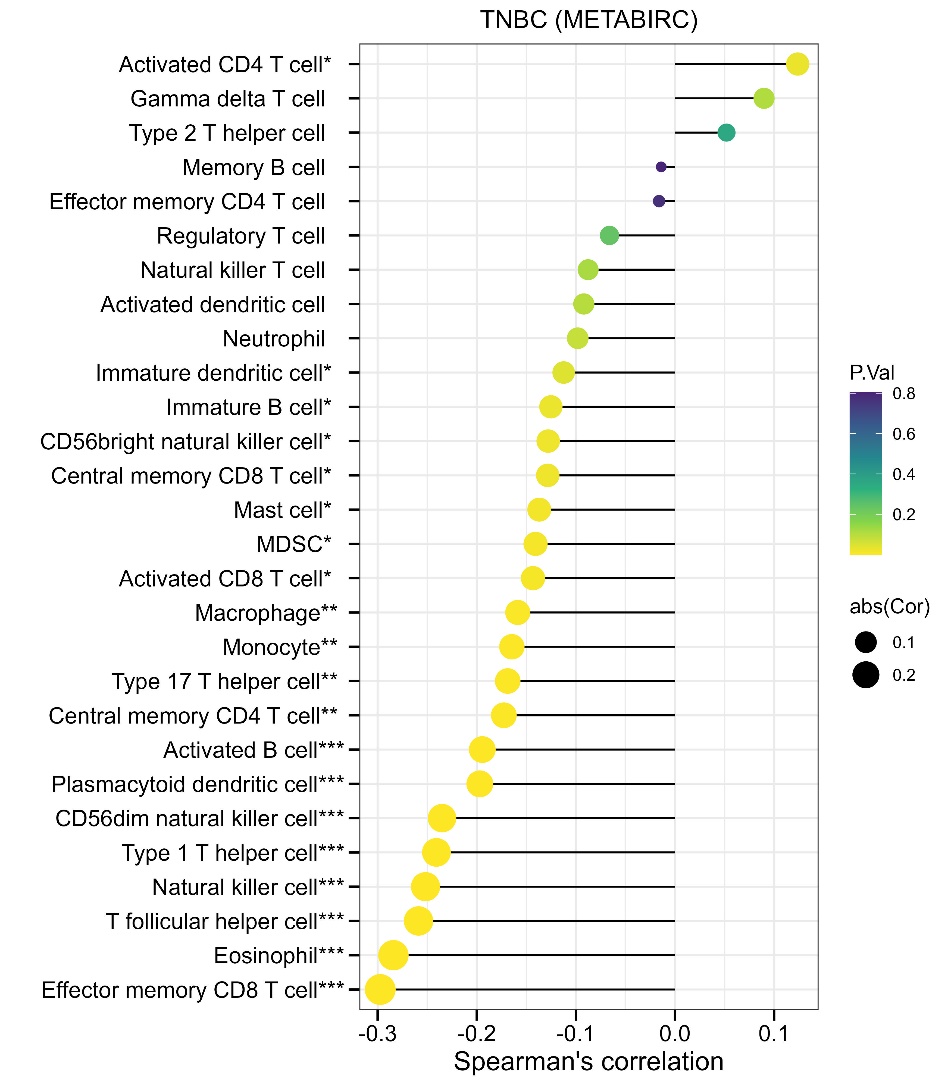


**Supplementary Figure 9.** Relationship between KNSTRN and immune cells in TNBC analyzed by ssGSEA.


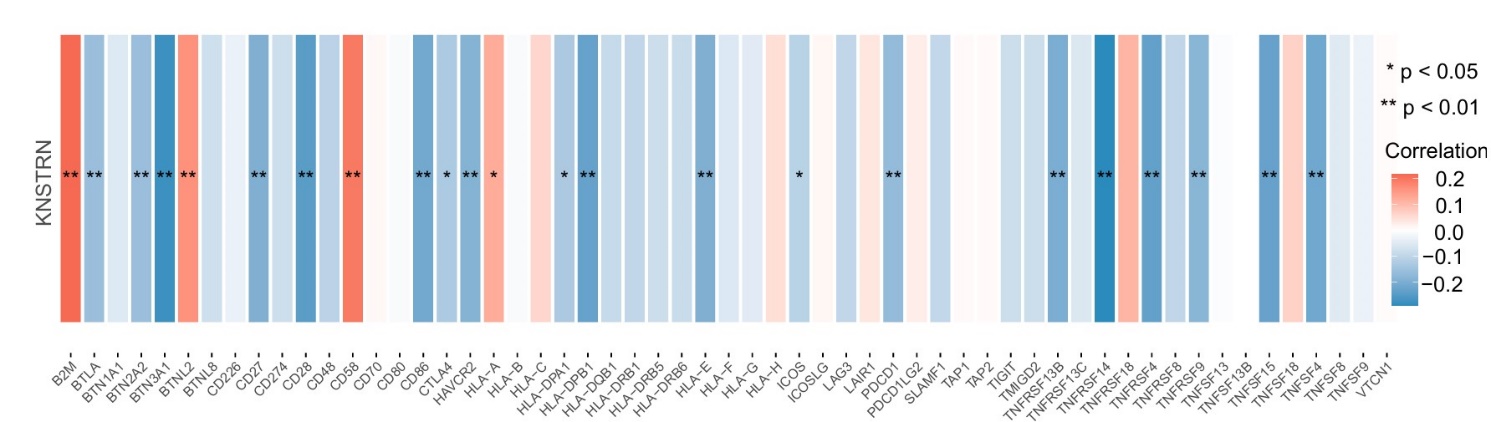
**Supplementary Figure 10.** Correlation between KNSTRN and immune-related gene expression in METABRIC RNA-seq dataset.

**
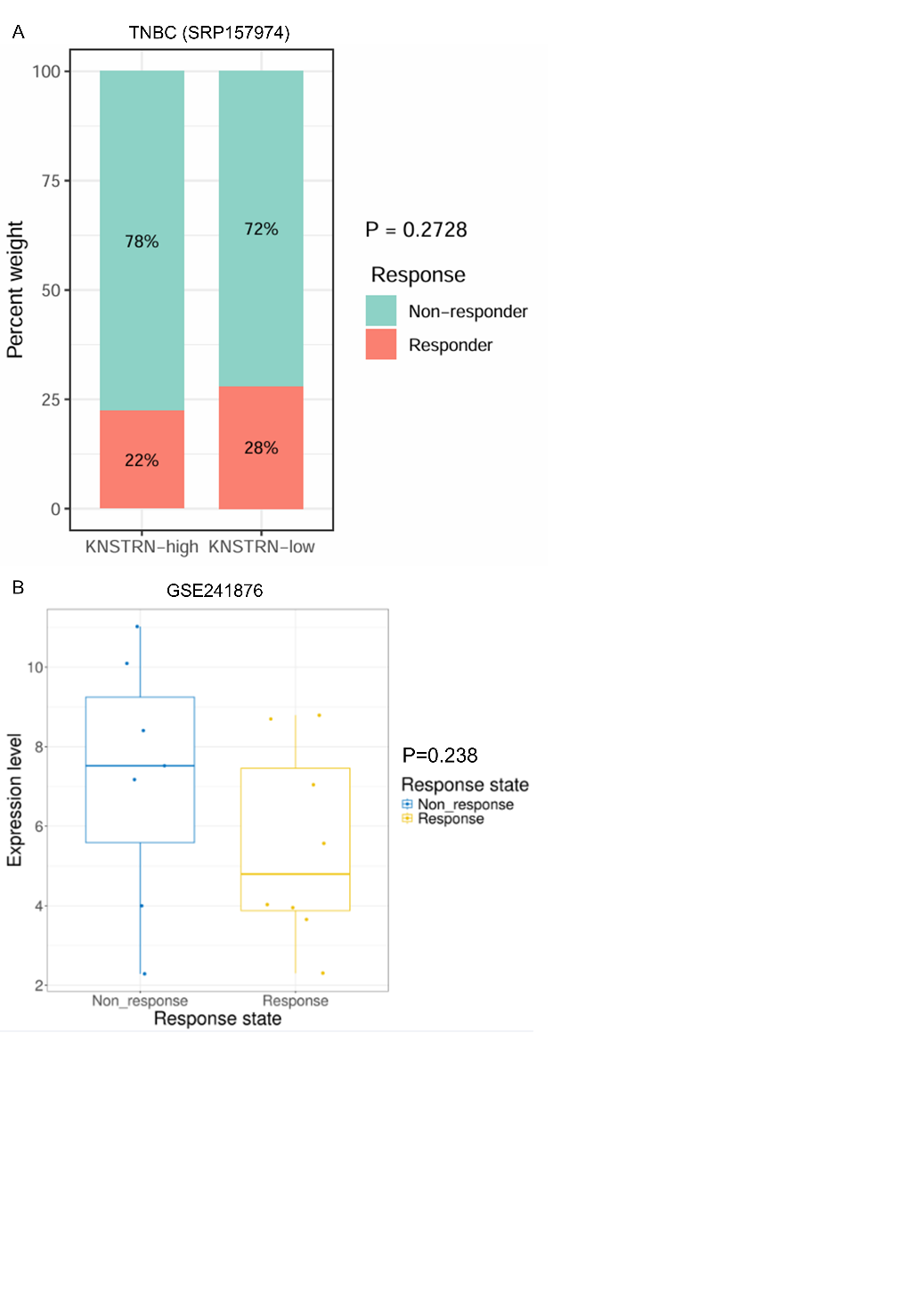
**

**Supplementary Figure 11. Evaluation of the predictive value of KNSTRN for the response to immunotherapy.**


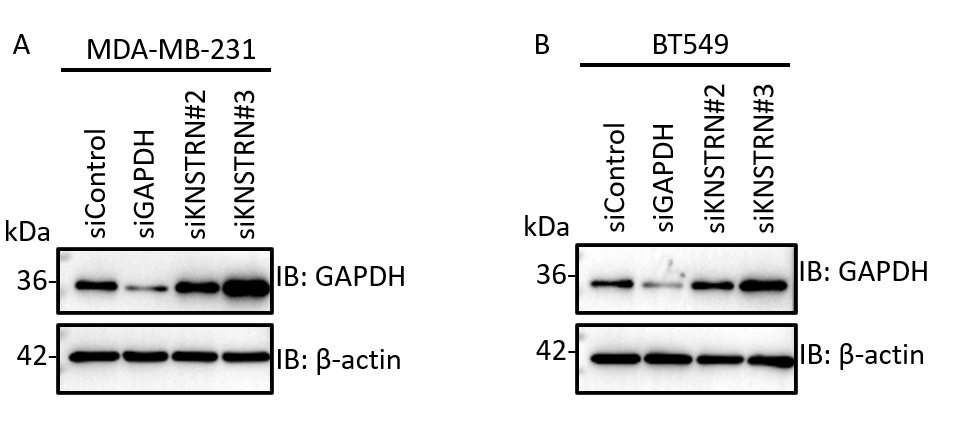


**Supplementary Figure 12**. The transfection efficiency of siGAPDH in MDA-MB-231. (A) and BT549 (B) cell lines were detected by Western blotting.
